# Gender-Specific Associations Between Sex Hormones and Cardiovascular Disease: A Systematic Review and Meta-Analysis

**DOI:** 10.31083/RCM47678

**Published:** 2026-05-13

**Authors:** Wenting Wang, Lulu Zhu, Miaomiao Qi, Jing Yu

**Affiliations:** ^1^Department of Cardiology, Lanzhou University Second Hospital, 730030 Lanzhou, Gansu, China

**Keywords:** gonadal steroid hormones, cardiovascular disease, sex factors, androgens, estrogens, postmenopause

## Abstract

**Background:**

Sex hormones play a critical role in the development of cardiovascular disease (CVD); however, the associations between specific circulating sex hormones and cardiovascular outcomes differ by sex, and the evidence remains inconclusive.

**Methods:**

A systematic review and meta-analysis of 23 prospective cohort studies was conducted to examine the associations between sex hormones and cardiovascular outcomes. Hazard ratios (HRs) with 95% confidence intervals (CIs) were extracted, and random- or fixed-effects models were applied based on heterogeneity. Subgroup analyses were performed by hormone type, age, and sex.

**Results:**

In men, higher total testosterone (TT) levels were associated with a reduced risk of CVD. No consistent associations were found between sex hormone-binding globulin (SHBG), calculated free testosterone (cFT), and estradiol with CVD. In women, elevated TT and cFT were linked to increased CVD risk. Estradiol exhibited a modest and uncertain protective effect. An outcome-specific analysis revealed that higher dehydroepiandrosterone (DHEA) levels were linked to an increased risk of heart failure, while SHBG was associated with reduced cardiovascular mortality. Conversely, higher estradiol concentrations correlated with increased cardiovascular mortality.

**Conclusions:**

Sex hormones exert complex, sex-specific, and outcome-dependent effects on cardiovascular risk. In men, higher testosterone levels are associated with lower overall CVD risk, whereas in women, these levels are linked to higher CVD and heart failure risk. Estradiol demonstrated a protective trend against major adverse cardiovascular events (MACEs), yet was linked to increased cardiovascular mortality. Higher SHBG levels are associated with lower cardiovascular mortality, whereas higher DHEA levels correlate with increased heart failure risk. Together, these findings underscore the importance of integrating sex, age, and endocrine context into cardiovascular risk stratification and prevention strategies.

**The PROSPERO Registration:**

CRD42024551055, Registration Link: https://www.crd.york.ac.uk/PROSPERO/view/CRD42024551055.

## 1. Introduction

Cardiovascular disease (CVD) is the leading cause of morbidity and mortality globally, responsible for an estimated 17.3 million deaths annually, with projections indicating an increase to 23.3 million by 2030 [1, 2]. Historically, CVD has been considered predominantly a male-dominant disease. However, contemporary evidence reveals that women, particularly postmenopausal women, face a comparable or even greater cardiovascular burden than men. Sex hormones have emerged as independent modulators of cardiovascular health, potentially explaining these sex-related differences in risk [3].

Female-specific conditions, such as pregnancy-related complications, polycystic ovary syndrome (PCOS), and premature menopause, are significant contributors to elevated cardiovascular risk and adverse long-term outcomes [4, 5]. Premenopausal women typically show a lower incidence of CVD compared to age-matched men [6]. However, this protection diminishes or reverses after menopause, suggesting hormonal dysregulation as a critical mechanism. In response to these disparities, the American Heart Association (AHA) issued its first clinical guidelines for the prevention of CVD in women in 1999, marking an important milestone in raising awareness of sex-specific cardiovascular risk [7]. Despite these efforts, significant gender-based differences in risk factors and disease prevalence persist, highlighting the continued need for tailored prevention and treatment strategies.

Emerging evidence highlights the complex relationship between estrogen exposure and cardiovascular outcomes. In women, the severity of coronary artery disease (CAD) is more strongly correlated with the duration of the postmenopausal period and age at menopause than with chronological age [8, 9]. A 20-year cohort study further found an inverse association between age at menopause and CVD mortality, suggesting that later menopause is associated with a reduced cardiovascular risk [10]. These findings highlight the critical role of declining ovarian function and reduced estrogen levels in influencing postmenopausal cardiovascular risk. Although men generally experience an earlier onset of CVD, the cumulative lifetime risk ultimately converges between sexes.

Despite these insights, the effects of specific sex hormones on cardiovascular outcomes remain controversial. To address this gap, a systematic review and meta-analysis of prospective cohort studies was conducted to evaluate the relationships between sex hormones and cardiovascular outcomes, with a focus on gender-specific differences. This study aims to provide comprehensive evidence on the impact of sex hormones on CVD, informing strategies for precision prevention and management in both men and women.

## 2. Materials and Methods

This study adhered to the Meta-Analysis of Observational Studies in Epidemiology (MOOSE) protocol. The search strategy formulation, establishment of inclusion and exclusion criteria, and execution of statistical analyses were conducted following the guidelines of the Preferred Reporting Items for Systematic Reviews and Meta-Analyses (PRISMA) [11, 12].

### 2.1 Literature Retrieval Strategy

**Fig. 1.**
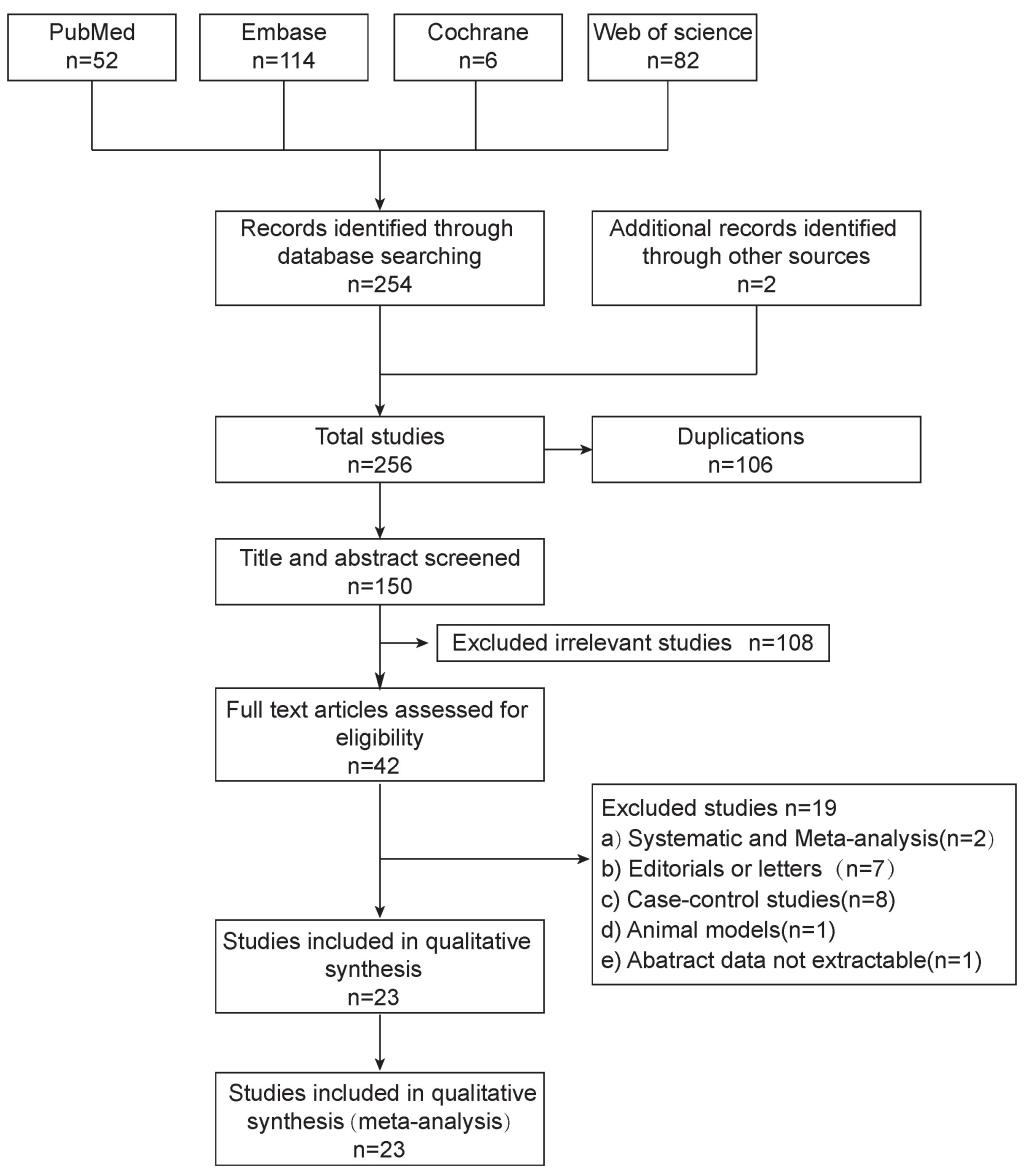
**Flow diagram**. Flowchart of study selection according to PRISMA guidelines.

A comprehensive literature search was performed across the PubMed, Embase, Cochrane Library, and Web of Science databases, covering articles published from the year 2000 to 2025. The search utilized the following terms: (cardiovascular disease OR heart failure OR coronary artery disease OR heart disease) AND (estradiol OR estrogen OR androgen OR testosterone OR sex hormone OR dehydroepiandrosterone) AND (cohort study). This search strategy was designed to identify studies aligned with the predefined inclusion criteria, ensuring a thorough and systematic review of the literature (Fig. 1).

### 2.2 Inclusion and Exclusion Criteria

#### 2.2.1 Inclusion Criteria

Studies eligible for inclusion were retrospective or prospective cohort studies investigating the relationship between sex hormones and CVD. Hazard ratios (HRs) and 95% confidence intervals (95% CIs) of sex hormones’ impact on CVD were extracted. There was no restriction on the language of publication during the literature search; however, all 23 cohort studies meeting the predefined inclusion criteria were published in English, and no non-English original articles were ultimately included.

#### 2.2.2 Exclusion Criteria

(a) Reviews, case reports, case-control studies, cross-sectional studies, letters, and other non-legible articles were excluded; (b) Studies that did not provide data suitable for meta-analysis were excluded.

### 2.3 Data Extraction and Statistical Analysis

#### 2.3.1 Pre-Specified Analyses and Exploratory Analyses

In accordance with the PROSPERO-registered protocol, the primary analyses pre-specified in this study involved evaluating the associations of total testosterone (TT), calculated free testosterone (cFT), sex hormone–binding globulin (SHBG), dehydroepiandrosterone (DHEA), and estradiol (E2) with overall CVD risk, stratified by sex (female vs male) and age (<60 vs ≥60 years). Multivariable-adjusted HRs were used as the effect measures. These sex- and age-stratified associations between sex hormones and overall CVD were designated as pre-specified primary (confirmatory) analyses.

Additionally, several exploratory analyses were conducted to further investigate heterogeneity and generate new hypotheses. This included outcome-specific meta-analyses by CVD outcomes (heart failure, MACE, cardiovascular mortality, and all-cause mortality), subgroup analyses based on hormone assay technology, and pooled analyses restricted to postmenopausal women. These analyses were not pre-specified as primary hypothesis tests in the PROSPERO protocol, and their findings should be interpreted with caution, warranting further confirmation in future studies.

#### 2.3.2 Data Extraction

The title, primary author, publication year, region, mean age, sample size, study population, sex hormones exposure, sex hormones assay technologies, primary cardiovascular outcomes, duration of follow-up, HRs, and corresponding 95% CIs were systematically extracted and independently summarized by the principal investigator. HRs were preferentially extracted from models labeled by the authors as “fully adjusted”, “multivariable-adjusted”, or similar terms to minimize confounding (**Supplementary Table 1**). For studies using multiple assay methods or repeated hormone measurements, a single set of effect estimates is derived based on the baseline hormone levels or their own hormone categories. Therefore, “higher” versus “lower” exposure, relative to higher versus lower levels within each source population and assay system, and within-study standardized HRs were pooled rather than absolute hormone concentrations.

Stratification by menopausal status and age followed the grouping and reporting schemes of the original studies. Due to the lack of individual-level data on perimenopausal status and exact age at menopause, age and menopausal status could not be modeled as continuous variables. Studies in which menopausal status was unclear or included women with mixed menopausal status were uniformly classified as “mixed female populations”. The quality of all included cohort studies was assessed using the Newcastle–Ottawa Scale (NOS) [13] (**Supplementary Table 2**).

#### 2.3.3 Data Collation and Statistical Analysis

Heterogeneity across studies was assessed using Cochran’s Q (Chi-square) test, alongside *I*^2^ and τ^2^ statistics, with pooled effect estimates derived using random-effects models. Subgroup analyses, random-effects meta-regression analyses, and leave-one-out sensitivity analyses were applied to further investigate potential sources of heterogeneity and assess the robustness of the summary estimates. Publication bias was evaluated by constructing funnel plots and applying Egger’s regression test, restricted to major subgroups with at least eight studies.

Statistical analyses were conducted using R, version 4.4.1 (R Foundation for Statistical Computing, Vienna, Austria) with the meta package, ensuring a robust and standardized approach to data synthesis and interpretation.

## 3. Results

### 3.1 Characteristics of Included Studies

A total of 23 studies, involving 8,068,754 participants (7,792,150 men and 276,604 women), were included in this meta-analysis. The mean age of male participants ranged from 46.98 to 76.3 years, while the mean age of female participants ranged from 31 to 74 years (**Supplementary Fig. 1**). The first author, publication year, mean age, region, sample size, follow-up duration, hormones exposure, and outcomes are summarized in Table 1 (Ref. [14, 15, 16, 17, 18, 19, 20, 21, 22, 23, 24, 25, 26, 27, 28, 29, 30, 31, 32, 33, 34, 35, 36]). Additionally, assay technologies and the quality assessment of the included cohort studies are provided in **Supplementary Table 2**.

**Table 1.** **Characteristics of included cohort studies on sex hormones and cardiovascular outcomes**.

First author/Year	Mean age (years)	Region	Population/sex	Sample size	Follow-up (years)	Hormones exposure	Outcomes
Schederecker F, *et al*., 2020 [14]	Male (61.6) Female (63.1)	Germany	Men and mixed female populations	3080	8.7	SHBG, TT, cFT, DHT, E2	Cardiovascular mortality
Yeap BB *et al*., 2022 [15]	58.0 (M)	United Kingdom	Men	210,700	9	TT, SHBG, cFT	MACE/Heart failure
Islam RM *et al*., 2022 [16]	74.0 (F)	Australia	Postmenopausal women	9180	4.5	DHEA, TT,	MACE
Zhao D *et al*., 2018 [17]	64.9 (F)	United States	Postmenopausal women	2834	12.1	TT, cFT, DHEA, SHBG, E2	CVD/Heart failure
Collet TH *et al*., 2020 [18]	72.4 (M)	United States	Men	552	7.4	TT, E2, SHBG	CVD
Meun C *et al*., 2018 [19]	70.19 (F)	Netherlands	Postmenopausal women	2578	11.36	TT, SHBG, DHEA	CVD
Yeap BB *et al*., 2014 [20]	76.2 (M)	Australia	Men	12,203	7.1	TT, cFT, DHT, E2	Heart failure
Zhao D *et al*., 2020 [21]	Male (63.2)	United States	Men and Postmenopausal women	8946	9.1	TT, DHEA, SHBG	Heart failure
	Female (62.8)						
Wang A *et al*., 2021 [22]	64.0 (F)	Sweden	Postmenopausal Women	2848	19.2	TT, cFT. SHBG	CVD/Heart failure
Pascual-Figal DA *et al*., 2009 [23]	53.1 (M)	Spain	Men	104	6.1	SHBG	Cardiovascular mortality
Laughlin GA *et al*., 2010 [24]	73.8 (F)	United States	Postmenopausal women	639	12.3	TT	CAD
Chen Y *et al*., 2011 [25]	60.2 (F)	United States	Postmenopausal women	99	19	E2, SHBG	CAD
Yeap BB *et al*., 2021 [26]	58.0 (M)	United Kingdom	Men	149,436	11.3	TT, SHBG, cFT	Cardiovascular mortality
Lim J *et al*., 2024 [27]	Male (56.32)	United Kingdom	Men and Postmenopausal women	358,036	12.5	TT, E2	Heart failure
Tuorila K *et al*., 2024 [28]	31/46 (F)	Finland	Pre-menopausal Women	5889	22	TT, SHBG, cFT	CVD
Harris K *et al*., 2023 [29]	Male (58)	United Kingdom	Men and Postmenopausal women	479,797	12.5	TT, SHBG, cFT	MI
	Female (557)						
Zhan X *et al*., 2024 [30]	46.98 (M)	United States	Men	6841	11	TT	Heart failure
Hsu B *et al*., 2016 [31]	76.9 (M)	Australia	Men	1705	5	TT, DHT, cFT, SHBG, E2	Cardiovascular mortality
Chan YX *et al*., 2016 [32]	50.3 (M)	Australia	Men	1804	14.9	TT, cFT, DHT, E2	CVD
Shores MM *et al*., 2014 [33]	76.3 (M)	United States	Men	1032	9	TT, cFT, DHT,	CVD
Ohlsson C *et al*., 2011 [34]	75.4 (M)	Sweden	Men	2416	5.1	TT, SHBG, cFT, E2	MACE
Khaw KT *et al*., 2007 [35]	57 (M)	United Kingdom	Men	1489	7	TT	Cardiovascular mortality
Arnlov J *et al*., 2006 [36]	56 (M)	United States	Men	2048	10	TT, DHEA, E2	CVD

Footnote: TT, total testosterone; SHBG, sex hormone-binding globulin; cFT, calculated free testosterone; E2, estradiol; DHEA, dehydroepiandrosterone; DHT, dihydrotestosterone; MACE, major adverse cardiovascular events; CVD, Cardiovascular disease; CAD, coronary artery disease; MI, myocardial infarction.

### 3.2 Effects of Sex Hormones on CVD in Male Patients

**Fig. 2.**
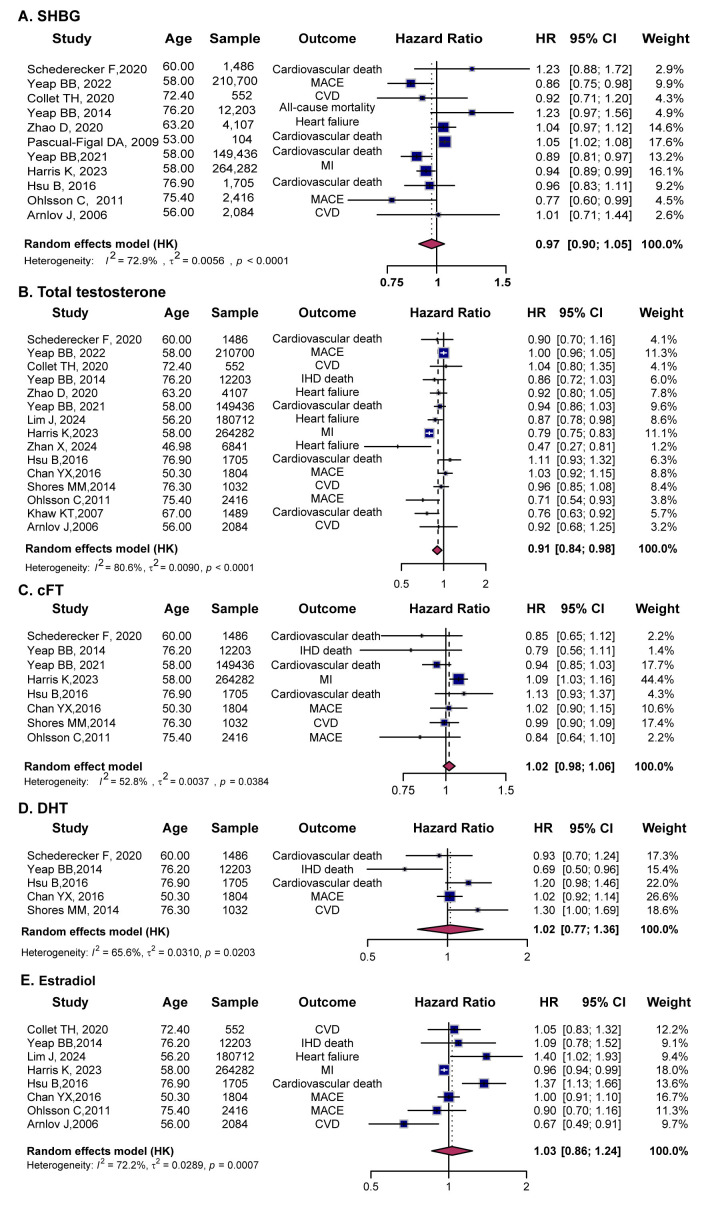
**Forest plots showing the associations between sex hormones and cardiovascular outcomes in males**. (A) SHBG, (B) TT, (C) cFT, (D) DHT, and (E) E2. Squares represent individual study HRs with 95% CIs, and diamonds indicate pooled estimates from random-effects models. SHBG, sex hormone-binding globulin; TT, total testosterone; cFT, calculated free testosterone; DHT, dihydrotestosterone; E2, Estradiol.

According to the pre-specified primary analysis plan, the association between sex hormones and overall CVD risk in men was first quantified. Higher TT levels were associated with a 9% reduction in CVD risk (HR = 0.91, 95% CI (0.84, 0.98)) (Fig. 2). Given the age-related variability in sex hormone levels, we conducted subgroup analyses stratified by age (<60 vs ≥60 years), a threshold commonly used in cardiovascular epidemiology and clinical practice to distinguish populations at lower and higher risk.

Ten studies evaluated the relationship between SHBG and CVD risk in men. The pooled analysis showed no significant relationship (HR = 0.97, 95% CI (0.90, 1.05)), with moderate heterogeneity (*I*^2^ = 72.9) (Fig. 2A). Age-stratified analysis revealed no significant association between SHBG and CVD risk in either the ≥60 or <60 years subgroup (≥60 or <60 years) (**Supplementary Fig. 2A**). Consistent with these findings, neither further stratification by 10-year age bands nor meta-regression using mean cohort age as a continuous moderator indicated that age accounted for the observed heterogeneity (**Supplementary Table 3**). Similarly, analyses stratified by assay technology and related meta-regression indicated that the measurement method did not contribute meaningfully to the observed heterogeneity. (**Supplementary Fig. 3A** and **Supplementary Table 4**).

A pooled analysis of ten studies assessing the relationship between TT and CVD risk indicated that higher TT levels were associated with a 9% reduction in CVD risk (HR = 0.91, 95% CI (0.84, 0.98)), though with substantial heterogeneity (*I*^2^ = 80.6) (Fig. 2B). Subgroup analyses confirmed consistent protective associations across age groups, with TT reducing CVD risk by 8% in men ≥60 years (HR = 0.92, 95% CI (0.86, 0.98)) and by 10% in men <60 years (HR = 0.90, 95% CI (0.82, 0.99)), suggesting a stable relationship regardless of age (**Supplementary Fig. 2B**). Meta-regression using 10-year age bands indicated that mean age accounted for virtually none of the between-study heterogeneity (**Supplementary Table 3**). In contrast, meta-regression by assay technology significantly reduced residual heterogeneity (*I*^2^ from 80.6% to 11.0%). These results suggest that the assay method is a key source of variability in the association of TT and CVD (**Supplementary Fig. 3B** and **Supplementary Table 5**).

The pooled analysis of eight studies showed no significant association between cFT and CVD risk (HR = 1.02, 95% CI (0.98, 1.06)), with modest heterogeneity (*I*^2^ = 52.8) (Fig. 2C). Subgroup analyses stratified by age indicated that the association between cFT and CVD was consistent across age groups (**Supplementary Fig. 2C**). Additionally, meta-regression with 10-year age bands as a continuous moderator showed that age did not substantially modify the association between cFT and CVD (**Supplementary Table 3**).

Four studies examined the relationship between DHT and CVD. The pooled analysis found no significant association (HR = 1.02, 95% CI (0.77, 1.36)) (Fig. 2D). Although Yeap BB *et al*., 2014 [20] observed a 31% reduction in risk with higher DHT levels (HR = 0.69, 95% CI (0.50, 0.96)); the findings from the other three studies were not significant.

Eight studies assessed the association between E2 and CVD risk in men. The pooled results showed no significant association (HR = 1.03, 95% CI (0.86, 1.24)) (Fig. 2E). Age-stratified analyses revealed no clear association between higher E2 levels and CVD risk (**Supplementary Fig. 2E**). Consistent with this, meta-regression treating mean age as a continuous moderator showed that age did not significantly influence the E2 and CVD relationship (**Supplementary Table 3**). Furthermore, meta-regression with assay technology as a categorical moderator did not reach statistical significance; assay type accounted for approximately 22% of the heterogeneity (R^2^ = 21.8%). Overall, these results suggest a largely null association between estradiol and CVD risk, with some assay-related discrepancies between liquid chromatography–tandem mass spectrometry (LC–MS/MS)- and radioimmunoassay (RIA)-based measurements, which warrant confirmation in future studies (**Supplementary Table 6**).

### 3.3 Effects of Sex Hormones on CVD in Female Patients

**Fig. 3.**
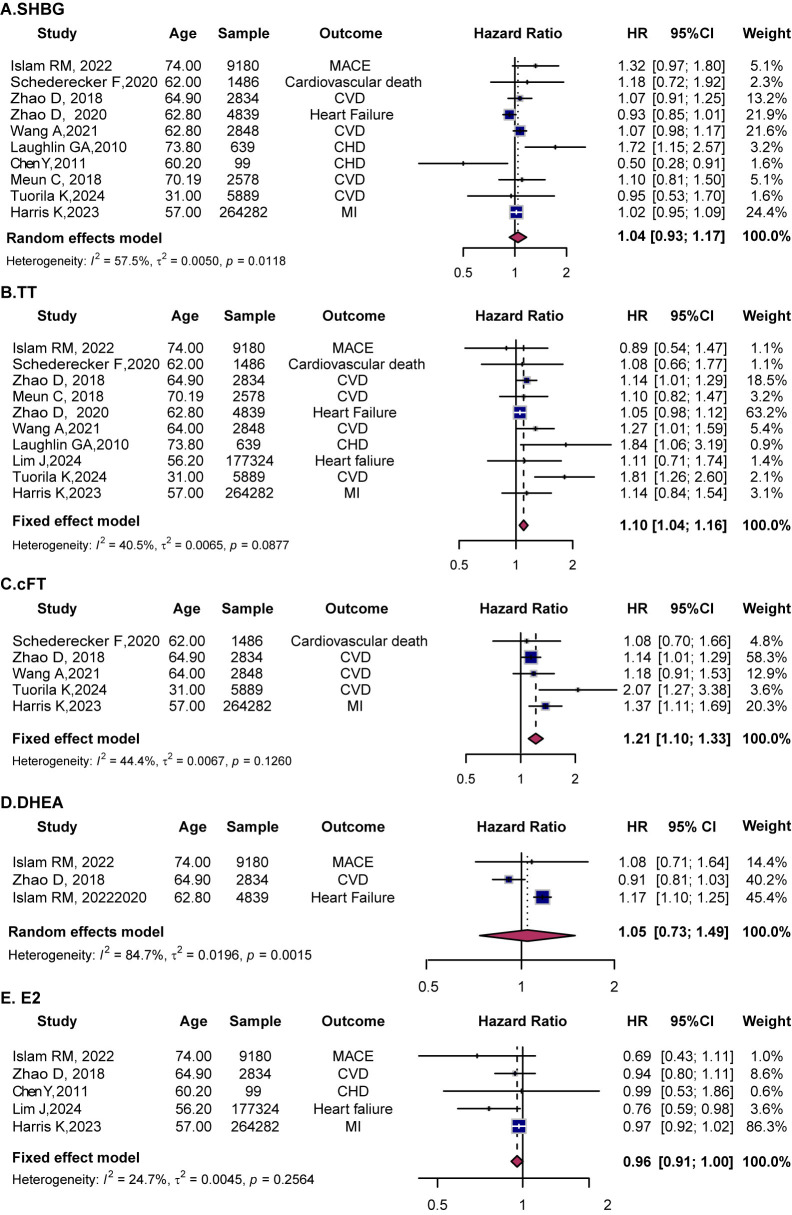
**Forest plots showing the associations between sex hormones and cardiovascular outcomes in females**. (A) SHBG, (B) TT, (C) cFT, (D) DHEA, (E) E2. HR with 95% CI are presented for each study (squares) and pooled estimates (diamonds) from random- or fixed-effects models. SHBG, sex hormone-binding globulin; TT, total testosterone; cFT, calculated free testosterone; DHEA, dehydroepiandrosterone; E2, estradiol.

According to the pre-specified primary analysis plan, a meta-analysis was conducted to pool the associations between each sex hormone and overall CVD risk in women. The pooled results indicated that higher TT and cFT were associated with a 10% (HR = 1.10, 95% CI (1.04, 1.16)) and 20% (HR = 1.21, 95% CI (1.10, 1.33)) increase in CVD risk, respectively. Additionally, higher E2 levels were associated with a borderline reduction in CVD risk (HR = 0.96, 95% CI (0.91, 1.00)) (Fig. 3).

Ten studies assessed the relationship between SHBG and CVD in women. In the overall analysis, SHBG was not significantly associated with CVD (HR = 1.04, 95% CI (0.93, 1.17)) (Fig. 3A), though substantial heterogeneity was observed (*I*^2^ = 57.5). Age-stratified analyses showed no significant associations in either subgroup (≥60 or <60 years) (**Supplementary Fig. 4A**). Further stratification by 10-year age bands indicated that higher SHBG levels were associated with increased CVD risk only in women aged 70–79 years (**Supplementary Table 3**). Meta-regression analyses incorporating mean age and assay technology as moderators indicated that neither variable significantly influenced the association between SHBG and CVD, nor did they account for the observed heterogeneity. (**Supplementary Table 7** and **Supplementary Fig. 5A** and **Supplementary Table 7**).

The pooled results of ten studies demonstrated a significant association between higher TT levels and increased CVD risk (HR = 1.10, 95% CI (1.04, 1.16), *I*^2^ = 40.5) (Fig. 3B). Age-stratified analyses indicated elevated risk in both older (≥60 years) and younger women (<60 years) (**Supplementary Fig. 4B**), and further stratification by 10-year age bands showed a significant association with low heterogeneity in the 60–69-year group (HR = 1.09, 95% CI (1.02, 1.17); *I*^2^ = 11.9%) (**Supplementary Table 3**). Random-effects meta-regression suggested that higher TT levels conferred greater excess CVD risk in relatively younger women (**Supplementary Table 8**), whereas assay technology did not significantly modify this association (**Supplementary Fig. 5B** and **Supplementary Table 8**).

Five studies assessed the relationship between cFT and CVD risk in women. The pooled results revealed a significant association with increased CVD risk HR = 1.21, 95% CI (1.10, 1.33), *I*^2^ = 44.4) (Fig. 3C). Subgroup analyses confirmed elevated in both women ≥60 and <60 years, with a stronger effect observed in the younger group (**Supplementary Fig. 4C**). Stratification by 10-year age bands further indicated that elevated cFT is associated with increased CVD risk across all age groups, with particularly robust and precise estimates in the 60–69-year group (**Supplementary Table 3**).

Three studies explored the association between DHEA and CVD risk in women. The pooled analysis found no significant association (HR = 1.05, 95% CI (0.73, 1.49)) (Fig. 3D). Additionally, five studies evaluated the role of E2 in female CVD risk. The pooled results suggested a borderline reduction in CVD risk (HR = 0.96, 95% CI (0.91, 1.00) *I*^2^ = 24.7) (Fig. 3E). Age-stratification analyses revealed no significant association between E2 levels and CVD risk in any age group (**Supplementary Fig. 4D**).

## 4. Effects of Sex Hormones on Different Cardiovascular Outcome Events

In exploratory outcome-specific analyses, the associations between sex hormones and heart failure, MACE, cardiovascular mortality, and all-cause mortality were assessed.

### 4.1 Effects of Sex Hormones on Heart Failure

**Fig. 4.**
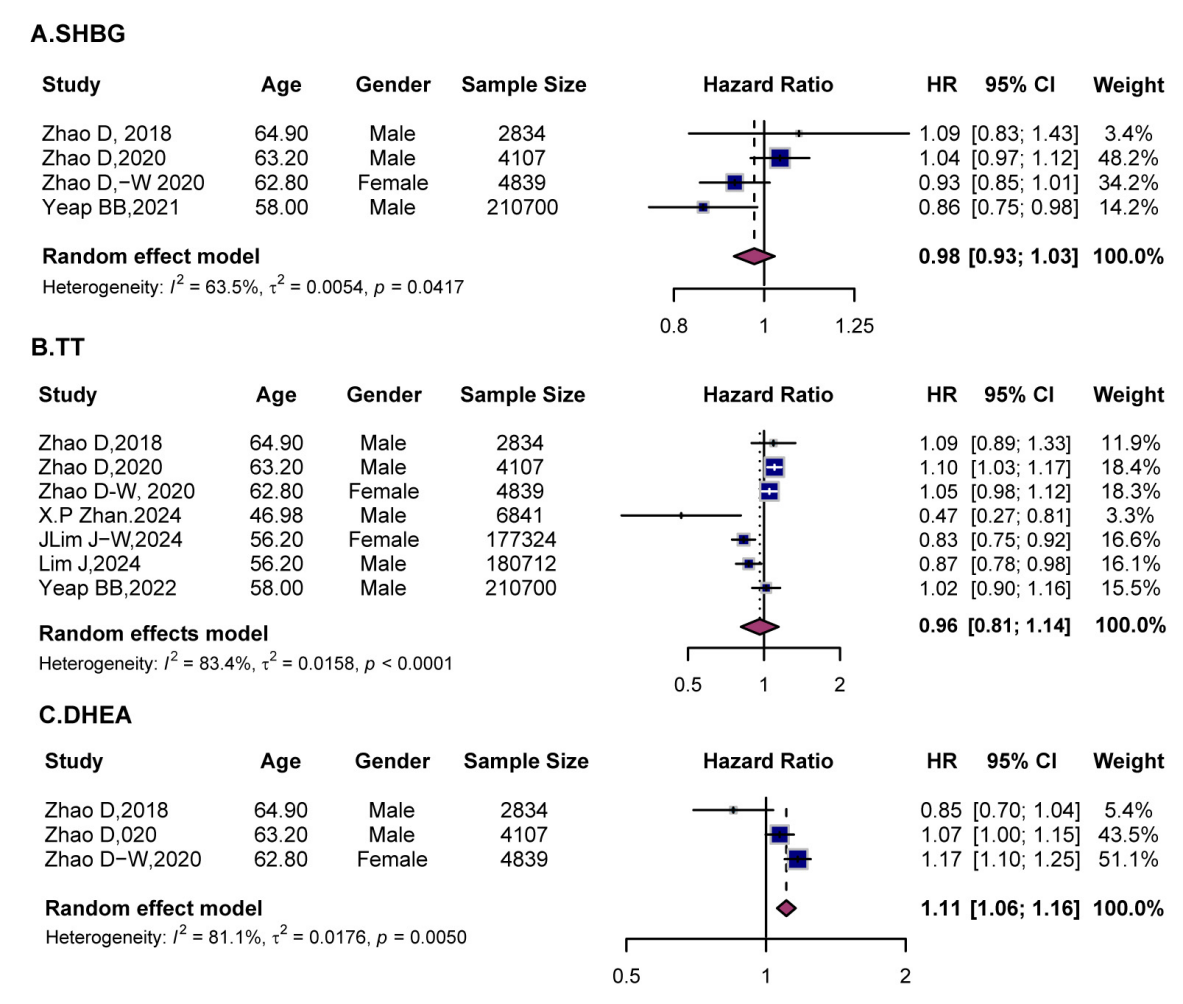
**Forest plots showing the associations between sex hormones and the risk of heart failure**. (A) SHBG, (B) TT, and (C) DHEA. Squares indicate HR with 95% CI for individual studies, and diamonds represent pooled estimates derived from random-effects models. SHBG, sex hormone-binding globulin; TT, total testosterone; DHEA, dehydroepiandrosterone.

A random-effects meta-analysis was conducted to evaluate the associations of SHBG, TT, and DHEA with heart failure risk. SHBG was not significantly associated with heart failure (HR = 0.98, 95% CI (0.93, 1.03)), while high DHEA levels were significantly associated with an increased risk (HR = 1.11, 95% CI (1.06, 1.16)) (Fig. 4).

For SHBG, the pooled analysis showed no overall association with heart failure (Fig. 4A). Subgroup analyses indicated a potential positive trend in women (Zhao *et al*., 2020 [21] and 2018 [17]), although the estimates were not statistically significant. In contrast, Yeap BB *et al*., 2021 [26] reported a protective effect (HR = 0.86, 95% CI (0.75, 0.98)). Sex- and age-stratified analyses revealed no significant effect modifications (**Supplementary Fig. 6A** and **7A**).

For TT, pooled estimates in men showed no significant association with heart failure (HR = 0.96, 95% CI (0.81, 1.14), *I*^2^ = 83.4) (Fig. 4B). Only two studies were available for women, with inconsistent results (**Supplementary Fig. 6B**). Notably, age-stratified analyses revealed an apparent “age paradox”. In individuals ≥60 years, higher TT levels consistently increased heart failure risk (HR = 1.08, 95% CI (1.03, 1.13)), whereas in those <60 years, TT was associated with a lower risk (HR = 0.88, 95% CI (0.83, 0.94)) (**Supplementary Fig. 7B**).

For DHEA, pooled results from three studies confirmed a significant positive association with heart failure (HR = 1.11, 95% CI (1.06, 1.16)) (Fig. 4C). Subgroup analyses revealed stronger effects in women (HR = 1.17) compared to men (HR = 1.07) (**Supplementary Fig. 6C**).

### 4.2 Effect of Sex Hormones on Major Adverse Cardiovascular Events (MACE)

**Fig. 5.**
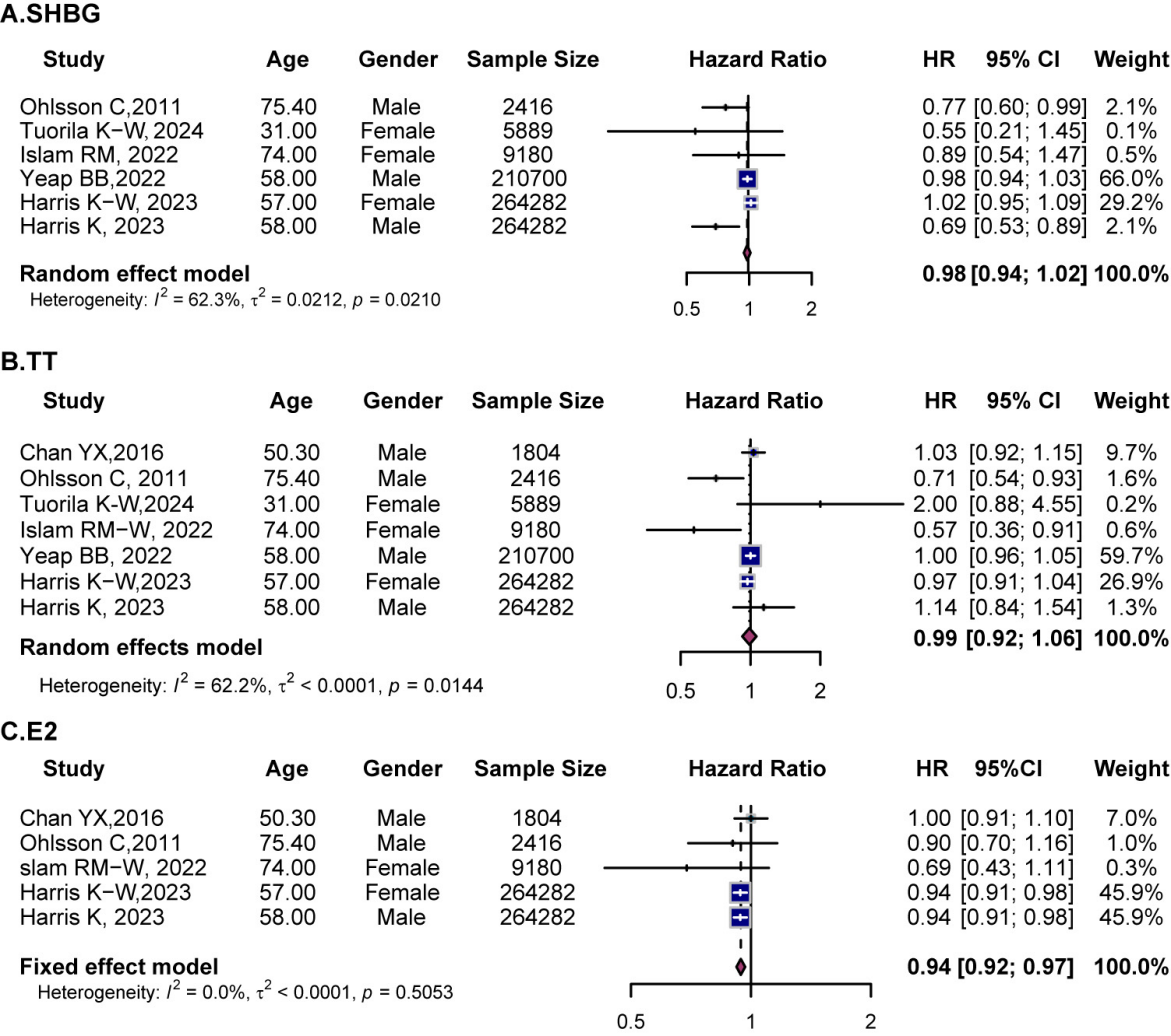
**Forest plots showing the associations between sex hormones and the risk of MACE**. (A) SHBG, (B) TT, (C) E2. Squares indicate HRs with 95% CI for individual studies, and diamonds represent pooled estimates derived from random-effects models. SHBG, sex hormone-binding globulin; TT, total testosterone; E2, Estradiol.

Meta-analysis of SHBG, TT, and E2 revealed heterogeneous associations with MACE risk (Fig. 5). For SHBG, pooled results demonstrated no significant association (HR = 0.98, 95% CI (0.94, 1.02)) (Fig. 5A). Subgroup analyses found no sex differences (**Supplementary Fig. 8A**). Age-stratified analyses showed no association in individuals ≥60 years (HR = 0.90, 95% CI (0.79, 1.10)), but a borderline protective trend in those <60 years (HR = 0.79, 95% CI (0.63, 1.00)) (**Supplementary Fig. 9A**).

For TT, pooled analysis indicated no overall association with MACE (HR = 0.99, 95% CI (0.92, 1.06)) (Fig. 5B). Subgroup analyses found no sex differences (**Supplementary Fig. 8B**). Age-stratified results showed neutral effects in individuals those ≥60 years (HR = 1.00, 95% CI (0.96, 1.03)), but significant protection in younger individuals <60 years (HR = 0.67, 95% CI (0.53, 0.85)) (**Supplementary Fig. 9B**).

For E2, fixed-effects analysis demonstrated a significant inverse association with MACE (HR = 0.94, 95% CI (0.92, 0.97)) (Fig. 5C). Results were robust with low heterogeneity. Protective effects were observed in both sexes (men, HR = 0.95, 95% CI (0.92, 0.98), women, HR = 0.94, 95% CI (0.90, 0.97)) (**Supplementary Fig. 8C**). Subgroup analyses indicated protection was mainly present in younger individuals, while null associations were found in those ≥60 years (**Supplementary Fig. 9C**).

### 4.3 Association Between Sex Hormones and Cardiovascular Mortality

**Fig. 6.**
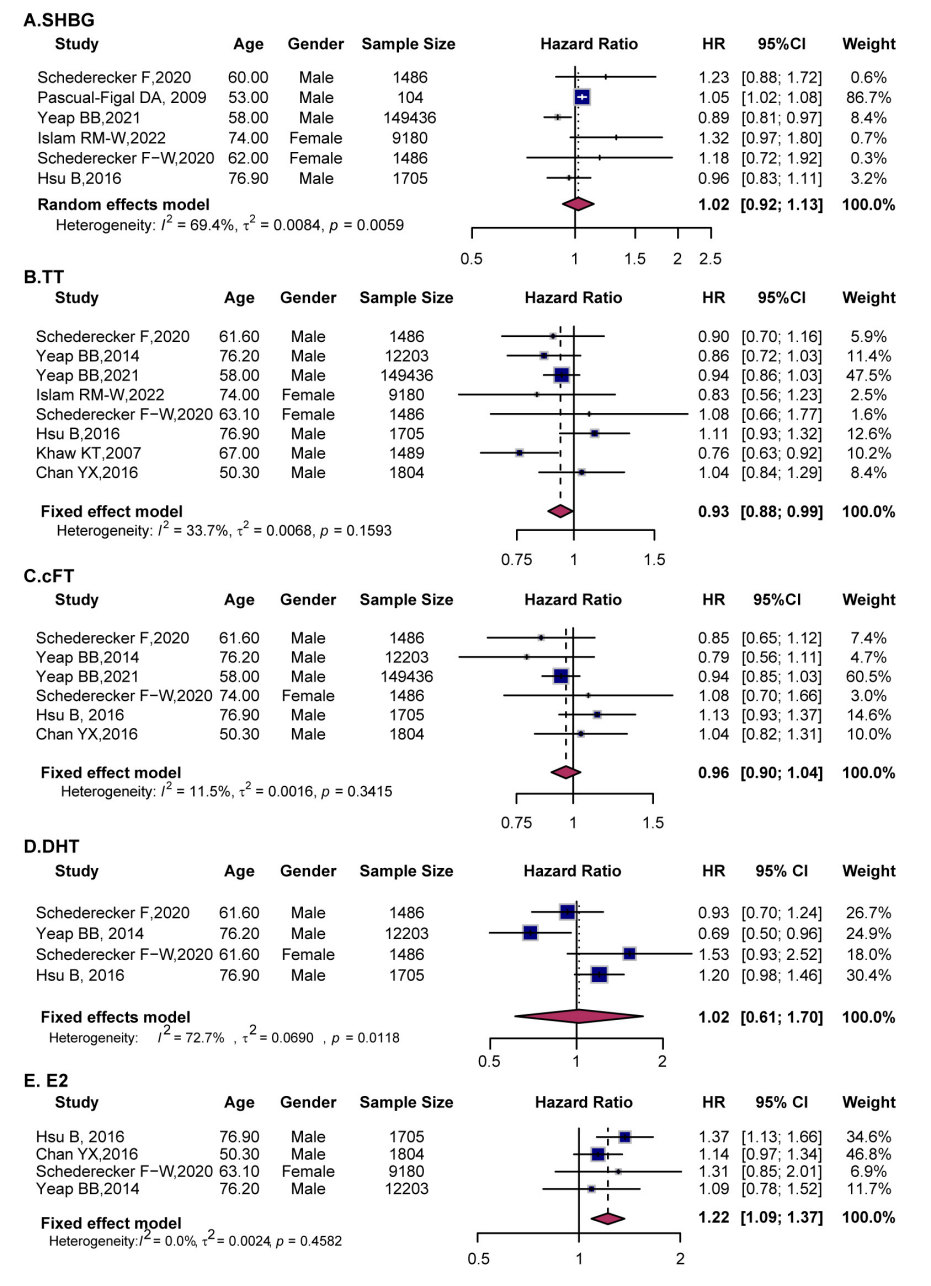
**Forest plots showing the associations between sex hormones and the risk of cardiovascular mortality**. (A) SHBG, (B) TT, (C) cFT, (D) DHT, and (E) E2. Squares indicate HRs with 95% CIs for individual studies, and diamonds represent pooled estimates derived from random-effects models. SHBG, sex hormone-binding globulin; TT, total testosterone; cFT, calculated free testosterone; DHT, dihydrotestosterone; E2, Estradiol.

The effects of SHBG, TT, cFT, DHT, and E2 on cardiovascular mortality were examined (Fig. 6). The pooled result indicated that circulating SHBG levels were not significantly associated with cardiovascular mortality (HR = 1.02, 95% CI (0.92, 1.13)) (Fig. 6A). Sex-stratified analyses (**Supplementary Fig. 10A**) revealed largely null associations between SHBG and cardiovascular mortality. Age-specific meta-analyses (<60 vs ≥60 years) did not demonstrate the relationship between SHBG and cardiovascular mortality (**Supplementary Fig. 11A**).

TT was significantly associated with a lower risk of cardiovascular mortality (HR = 0.93, 95% CI (0.88, 0.99)), particularly in men (HR = 0.94, 95% CI (0.88–1.00)), with low heterogeneity (Fig. 6B). Although age-stratified analyses did not reveal a clear association between TT and cardiovascular mortality (**Supplementary Fig. 11B**), sex-stratified analyses showed that higher TT levels were associated with a lower risk of cardiovascular death in both men and women (**Supplementary Fig. 10B**).

cFT showed no significant association (HR = 0.96, 95% CI (0.90, 1.04)) (Fig. 6C). The finding suggests that cFT is not significantly associated with cardiovascular mortality, and no evidence suggests that age or sex substantially modifies this relationship.

DHT demonstrated no significant association (HR = 1.02, 95% CI (0.61, 1.70)), with high heterogeneity across studies (Fig. 6D). In contrast, elevated E2 was robustly associated with increased cardiovascular mortality (HR = 1.22, 95% CI (1.09, 1.37)) (Fig. 6E). Age-stratified analyses showed significant positive associations in middle-aged and older individuals (**Supplementary Fig. 11E**), while sex-stratified analyses demonstrated a robust excess risk in men (**Supplementary Fig. 10E**), with a similar, though less precise, pattern in women. Overall, these results suggest that elevated estradiol is a relatively stable risk marker for cardiovascular death, regardless of age and sex.

### 4.4 Effect of Sex Hormones on All-Cause Mortality

**Fig. 7.**
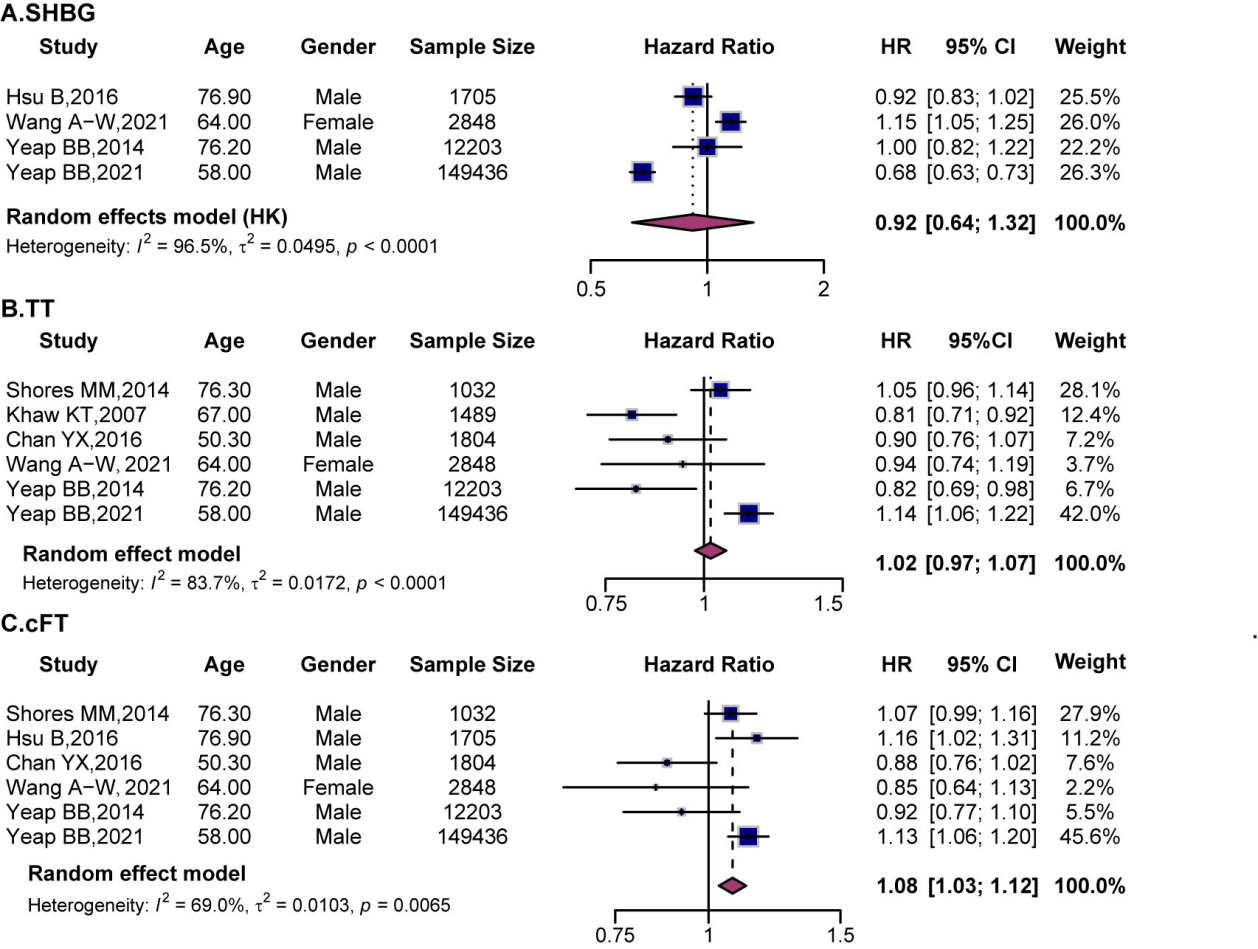
**Forest plots showing the associations between sex hormones and the risk of all-cause mortality**. (A) SHBG, (B) TT, and (C) cFT. Squares indicate HRs with 95% CIs for individual studies, and diamonds represent pooled estimates derived from random-effects models. SHBG, sex hormone-binding globulin; TT, total testosterone; cFT, calculated free testosterone.

This study analyzed the associations of SHBG, TT, and cFT with all-cause mortality (Fig. 7). The pooled results showed no significant associations for SHBG (HR = 0.92, 95% CI (0.64, 1.32)) or TT (HR = 1.02, 95% CI (0.97, 1.07)), while higher cFT levels were significantly associated with an 8% increase in all-cause mortality risk (HR = 1.08, 95% CI (1.03, 1.12)).

The pooled analysis showed no significant association between SHBG and all-cause mortality (HR = 0.92, 95% CI (0.64, 1.32)) (Fig. 7A). Subgroup analyses revealed no significant differences by sex (**Supplementary Fig. 12A**) or age (**Supplementary Fig. 13A**), suggesting inconsistent and heterogeneous findings. TT also showed no overall association (HR = 1.02, 95% CI (0.97, 1.07)), although study results varied widely (Fig. 7B). Subgroup analysis by sex found no significant association in men (HR = 0.95, 95% CI (0.82, 1.09)), while only one study assessed women and reported no significant effect (**Supplementary Fig. 12B**). Age-stratified analyses showed no differences across older and younger subgroups (**Supplementary Fig. 13B**).

cFT was significantly associated with an increased risk of all-cause mortality (HR = 1.08, 95% CI (1.03, 1.12)) (Fig. 7C). Subgroup analysis indicated that elevated cFT was associated with increased all-cause mortality in men (HR = 1.08, 95% CI (1.04, 1.13)). These results suggest that higher cFT levels are linked to increased all-cause mortality risk, with a more pronounced effect in men.

Additionally, in postmenopausal women, the associations between sex hormones and CVD were evaluated through pooled analyses. As shown in **Supplementary Fig. 14**, SHBG (HR = 0.98, 95% CI (0.93, 1.03)) and TT (HR = 1.03, 95% CI (0.90, 1.20)) were not significantly associated with CVD risk. cFT showed a non-significant trend toward elevated CVD risk (HR = 1.03, 95% CI (0.86, 1.24)), while higher E2 levels were associated with a slight reduction in CVD risk, though not statistically significant (HR = 0.98, 95% CI (0.87, 1.10)). These results suggest that relative androgen excess, particularly elevated TT and cFT, may predispose postmenopausal women to increased CVD risk, while SHBG and E2 do not display clear protective associations.

## 5. Discussion

This meta-analysis examined the associations between sex hormones and CVD risk, revealing notable sex-specific, age-dependent, and hormone type–specific differences. In men, higher TT levels were significantly associated with reduced CVD risk. This aligns with prior evidence linking low testosterone states to adverse health conditions, including type 2 diabetes [37], non-alcoholic fatty liver disease [38], CAD, and chronic obstructive pulmonary disease [39]. Low androgen levels may indicate overall frailty or systemic metabolic impairment [40]. In elderly men, both low TT and cFT independently predict cardiovascular events and mortality [40]. This observation is consistent with our finding that higher TT levels correlate with a reduced risk of cardiovascular mortality. Since low cFT often coexists with abnormal SHBG levels, this hormone pattern may exacerbate metabolic dysfunction and elevate CVD risk [41]. These data, together with the complex interactions among TT, cFT, and SHBG, emphasize the need for mechanistic and population-specific studies to distinguish causal effects from the impact of underlying metabolic frailty.

In women, the pattern is reversed. Elevated TT and cFT levels are significantly associated with an increased CVD risk. This sex divergence may reflect the loss of estrogen’s protective influence and the unopposed actions of androgens on vascular and cardiac remodeling [42, 43, 44]. Observational studies have shown that elevated TT is an independent risk factor for CVD and subclinical atherosclerosis in postmenopausal women [45]. Higher TT levels are associated with blood pressure variability and left ventricular hypertrophy [46]. However, the association between TT and CVD in postmenopausal women is not uniform [47]. Differences in population characteristics, including the prevalence of obesity and metabolic syndrome, may contribute to this heterogeneity [48]. Additionally, in postmenopausal women, the biological effects of androgens depend not only on total TT levels but also on SHBG and cFT, which together regulate androgen bioavailability [49, 50]. The relationship between TT and CVD risk is unlikely to be strictly linear and may involve threshold effects [24]. Such nonlinearity could partially explain the variability in both the strength and direction of the association across studies. Overall, these findings highlight a pronounced sex dependence in the relationship between TT and CVD risk.

SHBG, the primary transport protein for sex steroids, exerts complex, context-dependent effects. Low SHBG often coexists with obesity, insulin resistance, and dyslipidemia [51], all of which are well-established drivers of CVD [41]. Conversely, high SHBG, by lowering free testosterone, has also been identified as an independent predictor of CVD in certain male cohorts [40, 52]. In women, SHBG’s role is strongly influenced by estrogen. In premenopausal women with sufficient estrogen levels, elevated SHBG may serve as a marker of favorable metabolic health and exert indirect protective effects through mechanisms such as anti-inflammatory regulation [53] and improved insulin sensitivity [54]. However, in postmenopausal women with significantly reduced estrogen, high SHBG may further decrease bioavailable estrogen, thereby attenuating cardioprotective effects [19]. In metabolic disorders such as PCOS, low SHBG is a marker of disease severity and heightened CVD risk, further emphasizing its role as a regulator of metabolic and inflammatory burdens [55]. These observations suggest that the cardiovascular impact of SHBG and sex steroids must be interpreted within the broader context of endocrine-metabolic and therapeutic factors, rather than in isolation.

Recent data from hormone replacement therapy (HRT) trials support this context-dependent view. In postmenopausal women, the clinical significance of hormone concentrations is influenced by the surrounding endocrine–metabolic environment [56]. The associations between endogenous sex hormones and CVD risk observed in this study likely reflect the combined influence of hormone levels, obesity/metabolic phenotype, systemic inflammation, and HRT exposure [57]. Different HRT regimens and routes of administration also confer distinct cardiovascular and thrombotic risks [58]. These findings highlight the importance of considering the interactions among hormonal environment, metabolic status, and treatment exposure, and they caution against interpreting CVD risk based solely on serum sex hormone levels.

Elevated circulating DHEA showed no significant association with overall CVD risk, but was significantly linked to an increased risk of heart failure. DHEA, an adrenal-derived weak androgen that can be converted into more potent androgens, may have deleterious cardiovascular effects, including promoting cardiac hypertrophy [59, 60], driving adverse ventricular remodeling [61, 62], and inducing metabolic dysregulation [63]. The testosterone and DHEA may offer vaso-protective and anti-inflammatory benefits, but excessive or prolonged exposure can lead to mitochondrial dysfunction and maladaptive cardiac remodeling [64, 65]. These findings suggest that DHEA and testosterone exert a context-dependent “double-edged sword” effect on the heart. It should be noted that the specific mechanisms underlying this dual effect are inferred from and consistent with prior experimental and clinical studies. They are presented here as plausible mechanistic interpretations, rather than as conclusions directly demonstrated by the present meta-analysis.

Although estrogen is traditionally considered cardioprotective [66, 67], its effects appeared attenuated and heterogeneous in this study [68]. Overall, CVD and MACE are primarily composed of non-fatal myocardial infarction, non-fatal stroke, and coronary events requiring hospitalization, which typically represent earlier or intermediate stages of atherosclerotic disease. In these stages, moderately higher estradiol levels may offer protection by improving endothelial function, promoting vasodilation, optimizing lipid metabolism, and suppressing inflammation [69, 70, 71]. This aligns with the inverse associations between estradiol and overall CVD and MACE observed in women. In contrast, cardiovascular mortality is more often indicative of late-stage disease in older individuals with a high burden of comorbidities. Elevated estradiol levels are more likely to reflect an adverse endocrine–metabolic environment than a truly protective effect [72, 73]. Additionally, the cohort in the estradiol–MACE analyses was predominantly middle-aged to older individuals or early high-risk populations with mostly non-fatal events and relatively low heterogeneity. Differences in population characteristics and measurement methods across studies [74, 75] may further explain inconsistencies in effect estimates. Therefore, the apparently “protective” versus “harmful” associations of estradiol with cardiovascular outcomes likely reflect heterogeneous effects across different disease stages, outcome types, and metabolic–inflammatory contexts rather than contradictory biological actions.

Age emerged as a critical effect modifier. Associations between testosterone and overall CVD risk were stronger in individuals <60 years but attenuated in older adults, likely due to the interplay between vascular aging, endocrine alterations, and comorbidities. This highlights the importance of considering both biological sex and life stage when evaluating hormonal influences on cardiovascular risk. Although the <60 vs ≥60 years threshold is reasonable, the age structures and timing of menopause are not fully consistent across studies; thus, age-related differences in effect estimates should be interpreted with caution and warrant further confirmation in future individual participant data meta-analyses.

Notably, all associations observed in this meta-analysis are derived from observational cohort studies, and therefore cannot establish a causal relationship between sex hormones and cardiovascular outcomes. While multivariable models adjusted for age, conventional cardiovascular risk factors, and, in most studies, measures of adiposity and glucose metabolism were preferentially extracted, residual confounding due to obesity, insulin resistance, and related “metabolically unhealthy” phenotypes remains unavoidable. This is particularly relevant in postmenopausal women, where elevated androgens and reduced SHBG often reflect adverse metabolic states characterized by visceral adiposity, insulin resistance, and systemic inflammation—factors that themselves significantly contribute to increased cardiovascular risk.

In summary, this study reveals the multifaceted and outcomes-dependent roles of sex hormones in cardiovascular health. These findings highlight the need for future research and clinical practice to account for sex, age, and endocrine context, thereby enabling more precise cardiovascular risk stratification and individualized preventive strategies.

## 6. Limitations and Expectations

This study has several limitations. First, the included studies were highly heterogeneous in terms of cardiovascular outcomes and sex hormone indices. While no significant publication bias was detected for outcomes with ≥8 eligible studies, small-study effects and selective reporting cannot be entirely ruled out (**Supplementary Figs. 15–21**). This is especially relevant for subgroups with small effect sizes or moderate-to-high heterogeneity, where the results should be interpreted with caution. Second, most of the studies included were observational cohorts, with considerable variability in covariate adjustment across studies. Key metabolic confounders, such as obesity and insulin resistance, could not be harmonized at the individual-participant level. Consequently, residual confounding, reverse causation, and selection bias are difficult to eliminate, and strict causal inferences cannot be drawn. The associations observed in this study should be regarded primarily as hypothesis-generating signals that require validation in future mechanistic and interventional studies.

Third, this study included only cohorts with Newcastle–Ottawa Scale (NOS) scores ≥8 and relatively complete reporting, which enhances the internal validity of the pooled estimates but may have led to the omission of smaller, methodologically weaker, or null studies. Therefore, potential selection and publication bias cannot be entirely excluded. Additionally, hormone assay methods, sampling time points, and reporting units varied across studies, and the lack of standardized measurement protocols induces uncertainty in exposure assessment. Definitions of menopausal status were also not fully consistent, and some cohorts included women with mixed menopausal statuses, which could have introduced misclassification and further methodological heterogeneity. Additionally, the confirmatory conclusions of this study are based mainly on the PROSPERO pre-specified sex- and age-stratified analyses of overall CVD. In contrast, multidimensional stratified analyses by specific outcome types, assay methods, and subgroups restricted to postmenopausal women are exploratory in nature, of lower evidential strength, and primarily intended to aid in the interpretation of heterogeneity and generating hypotheses. These findings require further validation in independent cohorts and individual participant data analyses.

Despite these limitations, this meta-analysis provides a relatively comprehensive synthesis of current evidence on sex- and age-specific associations between sex hormones and CVD. Future research should prioritize large, prospective cohort and interventional studies with standardized hormone measurement protocols and extended follow-up, in order to more accurately clarify causal relationships between the modulation of sex hormone levels—particularly hyperandrogenic states in postmenopausal women—and cardiovascular risk.

## 7. Conclusion

This meta-analysis demonstrates that sex hormones exert context-dependent effects on cardiovascular risk. Testosterone was associated with a lower risk of CVD in men but with a higher risk in women. Estradiol was associated with a reduced risk of MACE but an increased risk of cardiovascular mortality. SHBG and DHEA exhibited opposing effects, with higher SHBG levels associated with a reduced risk of cardiovascular mortality, while higher DHEA levels were associated with an increased risk of heart failure. These findings highlight the importance of incorporating sex, age, and endocrine context into cardiovascular risk assessment and prevention strategies.

## Availability of Data and Materials

All data generated or analyzed during this study are included in this published article and its supplementary information files. The raw datasets, original Western blot images, and additional experimental materials are available from the corresponding author upon reasonable request.

## Declaration of AI and AI-Assisted Technologies in the Writing Process

During the preparation of this work, the author used an artificial intelligence–based large language model tool (ChatGPT, GPT-5 mini) in order to polish the language and improve clarity and readability for an international audience. The tool was used solely for editing and optimizing the wording; it did not contribute to the study design, data collection, statistical analysis, or interpretation of the results. All scientific content was independently generated, verified, and approved by the authors, who take full responsibility for the accuracy and integrity of the manuscript

## References

[1] Mendis S, Davis S, Norrving B (2015). Organizational update: the world health organization global status report on noncommunicable diseases 2014; one more landmark step in the combat against stroke and vascular disease. *Stroke*.

[2] Mathers CD, Loncar D (2006). Projections of global mortality and burden of disease from 2002 to 2030. *PLoS Medicine*.

[3] Timmis A, Aboyans V, Vardas P, Townsend N, Torbica A, Kavousi M (2024). European Society of Cardiology: the 2023 Atlas of Cardiovascular Disease Statistics. *European Heart Journal*.

[4] Veltman-Verhulst SM, van Rijn BB, Westerveld HE, Franx A, Bruinse HW, Fauser BCJM (2010). Polycystic ovary syndrome and early-onset preeclampsia: reproductive manifestations of increased cardiovascular risk. *Menopause (New York, N.Y.)*.

[5] Ray JG, Vermeulen MJ, Schull MJ, Redelmeier DA (2005). Cardiovascular health after maternal placental syndromes (CHAMPS): population-based retrospective cohort study. *Lancet (London, England)*.

[6] Sattar N, Greer IA (2002). Pregnancy complications and maternal cardiovascular risk: opportunities for intervention and screening?. *BMJ (Clinical Research Ed.)*.

[7] Mosca L, Grundy SM, Judelson D, King K, Limacher M, Oparil S (1999). Guide to Preventive Cardiology for Women.AHA/ACC Scientific Statement Consensus panel statement. *Circulation*.

[8] Roger VL, Go AS, Lloyd-Jones DM, Benjamin EJ, Berry JD, Borden WB (2012). Heart disease and stroke statistics–2012 update: a report from the American Heart Association. *Circulation*.

[9] Saltiki K, Doukas C, Kanakakis J, Anastasiou E, Mantzou E, Alevizaki M (2006). Severity of cardiovascular disease in women: relation with exposure to endogenous estrogen. *Maturitas*.

[10] de Kleijn MJJ, van der Schouw YT, Verbeek ALM, Peeters PHM, Banga JD, van der Graaf Y (2002). Endogenous estrogen exposure and cardiovascular mortality risk in postmenopausal women. *American Journal of Epidemiology*.

[11] Liberati A, Altman DG, Tetzlaff J, Mulrow C, Gøtzsche PC, Ioannidis JPA (2009). The PRISMA statement for reporting systematic reviews and meta-analyses of studies that evaluate healthcare interventions: explanation and elaboration. *BMJ (Clinical Research Ed.)*.

[12] Page MJ, McKenzie JE, Bossuyt PM, Boutron I, Hoffmann TC, Mulrow CD (2021). The PRISMA 2020 statement: an updated guideline for reporting systematic reviews. *BMJ (Clinical Research Ed.)*.

[13] Stang A (2010). Critical evaluation of the Newcastle-Ottawa scale for the assessment of the quality of nonrandomized studies in meta-analyses. *European Journal of Epidemiology*.

[14] Schederecker F, Cecil A, Prehn C, Nano J, Koenig W, Adamski J (2020). Sex hormone-binding globulin, androgens and mortality: the KORA-F4 cohort study. *Endocrine Connections*.

[15] Yeap BB, Marriott RJ, Antonio L, Raj S, Dwivedi G, Reid CM (2022). Associations of Serum Testosterone and Sex Hormone-Binding Globulin With Incident Cardiovascular Events in Middle-Aged to Older Men. *Annals of Internal Medicine*.

[16] Islam RM, Bell RJ, Handelsman DJ, McNeil JJ, Nelson MR, Reid CM (2022). Associations between blood sex steroid concentrations and risk of major adverse cardiovascular events in healthy older women in Australia: a prospective cohort substudy of the ASPREE trial. *The Lancet. Healthy Longevity*.

[17] Zhao D, Guallar E, Ouyang P, Subramanya V, Vaidya D, Ndumele CE (2018). Endogenous Sex Hormones and Incident Cardiovascular Disease in Post-Menopausal Women. *Journal of the American College of Cardiology*.

[18] Collet TH, Ewing SK, Ensrud KE, Laughlin GA, Hoffman AR, Varosy PD (2020). Endogenous Testosterone Levels and the Risk of Incident Cardiovascular Events in Elderly Men: The MrOS Prospective Study. *Journal of the Endocrine Society*.

[19] Meun C, Franco OH, Dhana K, Jaspers L, Muka T, Louwers Y (2018). High Androgens in Postmenopausal Women and the Risk for Atherosclerosis and Cardiovascular Disease: The Rotterdam Study. *The Journal of Clinical Endocrinology and Metabolism*.

[20] Yeap BB, Alfonso H, Chubb SAP, Handelsman DJ, Hankey GJ, Almeida OP (2014). In older men an optimal plasma testosterone is associated with reduced all-cause mortality and higher dihydrotestosterone with reduced ischemic heart disease mortality, while estradiol levels do not predict mortality. *The Journal of Clinical Endocrinology and Metabolism*.

[21] Zhao D, Guallar E, Ballantyne CM, Post WS, Ouyang P, Vaidya D (2020). Sex Hormones and Incident Heart Failure in Men and Postmenopausal Women: The Atherosclerosis Risk in Communities Study. *The Journal of Clinical Endocrinology and Metabolism*.

[22] Wang A, Gerstein HC, Lee SF, Hess S, Paré G, Rydén L (2021). Testosterone and sex hormone-binding globulin in dysglycemic women at high cardiovascular risk: A report from the Outcome Reduction with an Initial Glargine Intervention trial. *Diabetes & Vascular Disease Research*.

[23] Pascual-Figal DA, Tornel PL, Nicolás F, Sánchez-Más J, Martínez MD, Gracia MR (2009). Sex hormone-binding globulin: a new marker of disease severity and prognosis in men with chronic heart failure. *Revista Espanola De Cardiologia*.

[24] Laughlin GA, Goodell V, Barrett-Connor E (2010). Extremes of endogenous testosterone are associated with increased risk of incident coronary events in older women. *The Journal of Clinical Endocrinology and Metabolism*.

[25] Chen Y, Zeleniuch-Jacquotte A, Arslan AA, Wojcik O, Toniolo P, Shore RE (2011). Endogenous hormones and coronary heart disease in postmenopausal women. *Atherosclerosis*.

[26] Yeap BB, Marriott RJ, Antonio L, Chan YX, Raj S, Dwivedi G (2021). Serum Testosterone is Inversely and Sex Hormone-binding Globulin is Directly Associated with All-cause Mortality in Men. *The Journal of Clinical Endocrinology and Metabolism*.

[27] Lim J, Hashemian M, Blechter B, Roger VL, Wong JYY (2024). Pre-diagnostic free androgen and estradiol levels influence heart failure risk in both women and men: A prospective cohort study in the UK Biobank. *European Journal of Heart Failure*.

[28] Tuorila K, Ollila MM, Hurskainen E, Tapanainen J, Franks S, Piltonen T (2024). Association of hyperandrogenaemia with hypertension and cardiovascular events in pre-menopausal women: a prospective population-based cohort study. *European Journal of Endocrinology*.

[29] Harris K, Peters SAE, Woodward M (2023). Sex hormones and the risk of myocardial infarction in women and men: a prospective cohort study in the UK Biobank. *Biology of Sex Differences*.

[30] Zhan X, Liu Y, Chen T, Wan H, Xiong S, Li S (2024). The association between serum testosterone level and congestive heart failure in US male adults: data from National Health and Nutrition Examination Survey (NHANES) 2011-2016. *Reproductive Biology and Endocrinology: RB&E*.

[31] Hsu B, Cumming RG, Naganathan V, Blyth FM, Le Couteur DG, Hirani V (2016). Temporal Changes in Androgens and Estrogens Are Associated With All-Cause and Cause-Specific Mortality in Older Men. *The Journal of Clinical Endocrinology and Metabolism*.

[32] Chan YX, Knuiman MW, Hung J, Divitini ML, Beilby JP, Handelsman DJ (2016). Neutral associations of testosterone, dihydrotestosterone and estradiol with fatal and non-fatal cardiovascular events, and mortality in men aged 17-97 years. *Clinical Endocrinology*.

[33] Shores MM, Biggs ML, Arnold AM, Smith NL, Longstreth WT, Kizer JR (2014). Testosterone, dihydrotestosterone, and incident cardiovascular disease and mortality in the cardiovascular health study. *The Journal of Clinical Endocrinology and Metabolism*.

[34] Ohlsson C, Barrett-Connor E, Bhasin S, Orwoll E, Labrie F, Karlsson MK (2011). High serum testosterone is associated with reduced risk of cardiovascular events in elderly men. The MrOS (Osteoporotic Fractures in Men) study in Sweden. *Journal of the American College of Cardiology*.

[35] Khaw KT, Dowsett M, Folkerd E, Bingham S, Wareham N, Luben R (2007). Endogenous testosterone and mortality due to all causes, cardiovascular disease, and cancer in men: European prospective investigation into cancer in Norfolk (EPIC-Norfolk) Prospective Population Study. *Circulation*.

[36] Arnlöv J, Pencina MJ, Amin S, Nam BH, Benjamin EJ, Murabito JM (2006). Endogenous sex hormones and cardiovascular disease incidence in men. *Annals of Internal Medicine*.

[37] Kautzky-Willer A, Harreiter J, Pacini G (2016). Sex and Gender Differences in Risk, Pathophysiology and Complications of Type 2 Diabetes Mellitus. *Endocrine Reviews*.

[38] Jaruvongvanich V, Sanguankeo A, Riangwiwat T, Upala S (2017). Testosterone, Sex Hormone-Binding Globulin and Nonalcoholic Fatty Liver Disease: a Systematic Review and Meta-Analysis. *Annals of Hepatology*.

[39] Ruige JB, Mahmoud AM, De Bacquer D, Kaufman JM (2011). Endogenous testosterone and cardiovascular disease in healthy men: a meta-analysis. *Heart (British Cardiac Society)*.

[40] Yeap BB (2010). Androgens and cardiovascular disease. *Current Opinion in Endocrinology, Diabetes, and Obesity*.

[41] Mesch VR, Siseles NO, Maidana PN, Boero LE, Sayegh F, Prada M (2008). Androgens in relationship to cardiovascular risk factors in the menopausal transition. *Climacteric: the Journal of the International Menopause Society*.

[42] Ziemens B, Wallaschofski H, Völzke H, Rettig R, Dörr M, Nauck M (2013). Positive association between testosterone, blood pressure, and hypertension in women: longitudinal findings from the Study of Health in Pomerania. *Journal of Hypertension*.

[43] Rexrode KM, Manson JE, Lee IM, Ridker PM, Sluss PM, Cook NR (2003). Sex hormone levels and risk of cardiovascular events in postmenopausal women. *Circulation*.

[44] Sohrabji F, Okoreeh A, Panta A (2019). Sex hormones and stroke: Beyond estrogens. *Hormones and Behavior*.

[45] Macut D, Antić IB, Bjekić-Macut J (2015). Cardiovascular risk factors and events in women with androgen excess. *Journal of Endocrinological Investigation*.

[46] Chen J, Wang Q, Pei Y, Li N, Han J, Yu J (2021). Effect of free androgen index on blood pressure variability and target organ damage in postmenopausal hypertensive women: findings from a cross-sectional study. *Menopause (New York, N.Y.)*.

[47] Luo X, Wang Y, Wang L, Shen Y, Ren M (2024). Association Between Female Androgen Levels, Metabolic Syndrome, and Cardiovascular Disease: An NHANES Analysis (2013-2016). *International Journal of Women’s Health*.

[48] Patel SM, Ratcliffe SJ, Reilly MP, Weinstein R, Bhasin S, Blackman MR (2009). Higher serum testosterone concentration in older women is associated with insulin resistance, metabolic syndrome, and cardiovascular disease. *The Journal of Clinical Endocrinology and Metabolism*.

[49] Janssen I, Powell LH, Crawford S, Lasley B, Sutton-Tyrrell K (2008). Menopause and the metabolic syndrome: the Study of Women’s Health Across the Nation. *Archives of Internal Medicine*.

[50] Olszanecka A, Kawecka-Jaszcz K, Czarnecka D (2016). Association of free testosterone and sex hormone binding globulin with metabolic syndrome and subclinical atherosclerosis but not blood pressure in hypertensive perimenopausal women. *Archives of Medical Science: AMS*.

[51] Wild RA (1995). Obesity, lipids, cardiovascular risk, and androgen excess. *The American Journal of Medicine*.

[52] Gyawali P, Martin SA, Heilbronn LK, Vincent AD, Jenkins AJ, Januszewski AS (2018). Cross-sectional and longitudinal determinants of serum sex hormone binding globulin (SHBG) in a cohort of community-dwelling men. *PloS One*.

[53] Yamazaki H, Kushiyama A, Sakoda H, Fujishiro M, Yamamotoya T, Nakatsu Y (2018). Protective Effect of Sex Hormone-Binding Globulin against Metabolic Syndrome: In Vitro Evidence Showing Anti-Inflammatory and Lipolytic Effects on Adipocytes and Macrophages. *Mediators of Inflammation*.

[54] Wallace IR, McKinley MC, Bell PM, Hunter SJ (2013). Sex hormone binding globulin and insulin resistance. *Clinical Endocrinology*.

[55] García-Sáenz MR, Ferreira-Hermosillo A, Lobaton-Ginsberg M (2022). Proinflammatory cytokines in polycystic ovarian syndrome. *Revista Medica Del Instituto Mexicano Del Seguro Social*.

[56] Mattioli AV (2025). Therapy, and Gout in Older Women: An Overlooked Connection: A Comment on “Comparison of Clinical Characteristics in Older-Onset and Common-Age-of-Onset Gout: A Prospective Gout Cohort Study” by Do et al. *Drugs & Aging*.

[57] Yanachkova V, Vasileva-Slaveva M, Kostov S, Yordanov A (2025). Reconsidering Hormone Replacement Therapy: Current Insights on Utilisation in Premenopausal and Menopausal Women: An Overview. *Journal of Clinical Medicine*.

[58] Foschi M, Groccia G, Rusce ML, Medaglia C, Aio C, Sponzilli A (2025). Estradiol and Micronized Progesterone: A Narrative Review About Their Use as Hormone Replacement Therapy. *Journal of Clinical Medicine*.

[59] Chen J, Yu J, Yuan R, Li N, Li C, Zhang X (2022). mTOR inhibitor improves testosterone-induced myocardial hypertrophy in hypertensive rats. *The Journal of Endocrinology*.

[60] Curl CL, Delbridge LMD, Canny BJ, Wendt IR (2009). Testosterone modulates cardiomyocyte Ca(2+) handling and contractile function. *Physiological Research*.

[61] Scott JM, Dillon EL, Kinsky M, Chamberlain A, McCammon S, Jupiter D (2019). Effects of adjunct testosterone on cardiac morphology and function in advanced cancers: an ancillary analysis of a randomized controlled trial. *BMC Cancer*.

[62] Goglia L, Tosi V, Sanchez AM, Flamini MI, Fu XD, Zullino S (2010). Endothelial regulation of eNOS, PAI-1 and t-PA by testosterone and dihydrotestosterone in vitro and in vivo. *Molecular Human Reproduction*.

[63] Pirompol P, Teekabut V, Weerachatyanukul W, Bupha-Intr T, Wattanapermpool J (2016). Supra-physiological dose of testosterone induces pathological cardiac hypertrophy. *The Journal of Endocrinology*.

[64] Chistiakov DA, Myasoedova VA, Melnichenko AA, Grechko AV, Orekhov AN (2018). Role of androgens in cardiovascular pathology. *Vascular Health and Risk Management*.

[65] Bianchi VE (2018). Testosterone, myocardial function, and mortality. *Heart Failure Reviews*.

[66] de Oliveira TS, de Oliveira LM, de Oliveira LP, Costa RMD, Tostes RDC, Georg RDC (2018). Activation of PI3K/Akt pathway mediated by estrogen receptors accounts for estrone-induced vascular activation of cGMP signaling. *Vascular Pharmacology*.

[67] Holder SM, Brislane Á, Dawson EA, Hopkins ND, Hopman MTE, Cable NT (2019). Relationship Between Endothelial Function and the Eliciting Shear Stress Stimulus in Women: Changes Across the Lifespan Differ to Men. *Journal of the American Heart Association*.

[68] Davezac M, Buscato M, Zahreddine R, Lacolley P, Henrion D, Lenfant F (2021). Estrogen Receptor and Vascular Aging. *Frontiers in Aging*.

[69] Iorga A, Cunningham CM, Moazeni S, Ruffenach G, Umar S, Eghbali M (2017). The protective role of estrogen and estrogen receptors in cardiovascular disease and the controversial use of estrogen therapy. *Biology of Sex Differences*.

[70] Miller VM, Duckles SP (2008). Vascular actions of estrogens: functional implications. *Pharmacological Reviews*.

[71] El Khoudary SR, Aggarwal B, Beckie TM, Hodis HN, Johnson AE, Langer RD (2020). Menopause Transition and Cardiovascular Disease Risk: Implications for Timing of Early Prevention: A Scientific Statement From the American Heart Association. *Circulation*.

[72] Maggio M, Ceda GP, Lauretani F, Bandinelli S, Ruggiero C, Guralnik JM (2009). Relationship between higher estradiol levels and 9-year mortality in older women: the Invecchiare in Chianti study. *Journal of the American Geriatrics Society*.

[73] Lau L, Wiebe N, Ramesh S, Ahmed S, Klarenbach S, Carrero JJ (2023). Associations of Estradiol With Mortality and Health Outcomes in Patients Undergoing Hemodialysis: A Prospective Cohort Study. *Canadian Journal of Kidney Health and Disease*.

[74] Cho L, Kaunitz AM, Faubion SS, Hayes SN, Lau ES, Pristera N (2023). Rethinking Menopausal Hormone Therapy: For Whom, What, When, and How Long?. *Circulation*.

[75] Mehta JM, Chester RC, Kling JM (2019). The Timing Hypothesis: Hormone Therapy for Treating Symptomatic Women During Menopause and Its Relationship to Cardiovascular Disease. *Journal of Women’s Health (2002)*.

