# Supplementary material for: Gender-Specific Associations Between Sex Hormones and Cardiovascular Disease: A Systematic Review and Meta-Analysis
Source: Rev Cardiovasc Med. 2026 May 13;27(5):47678. doi: 10.31083/RCM47678 (PMC13227368; doi:10.31083/RCM47678)
Supplement: Supplementary file 1 [file 2153-8174-27-5-47678-s1.zip › Supplementary Figures.docx]

**Supplementary Figures**


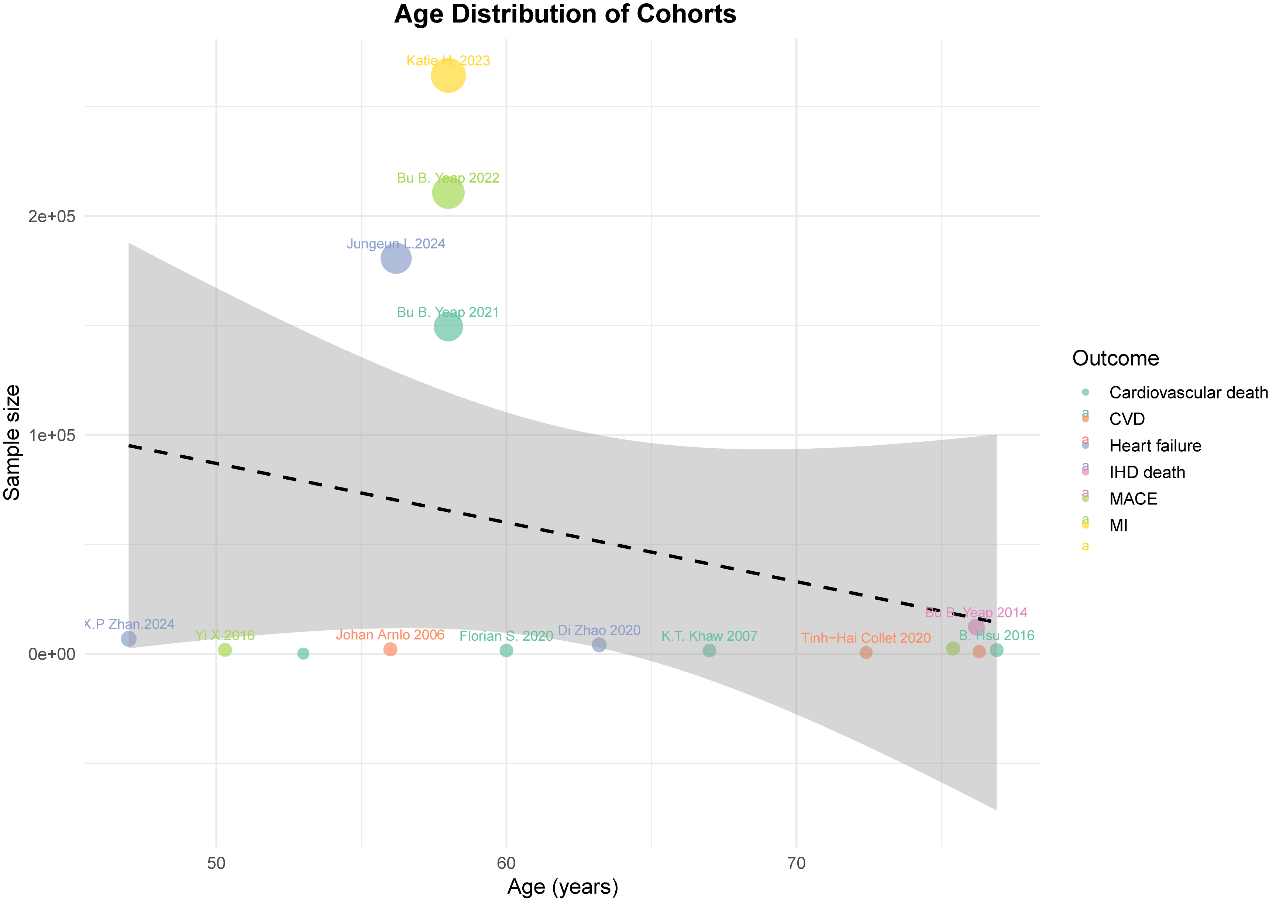


Supplementary Fig.1 Overview of the individual studies included in the meta-analysis, stratified by gender. The plot summarizes key characteristics of each study. The x-axis represents the mean age of participants. The y-axis lists the specific cardiovascular outcomes assessed. The size of each bubble corresponds to the sample size of that individual study.


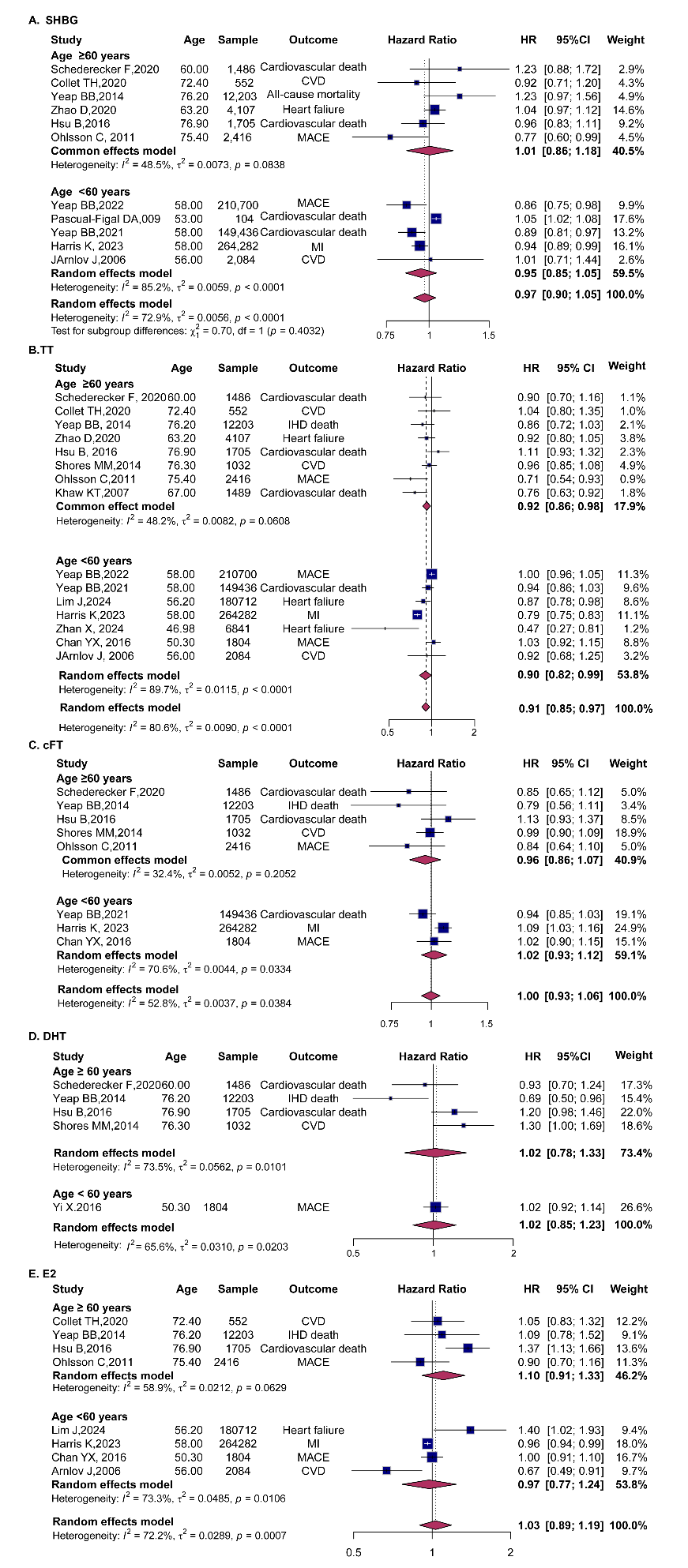
Supplementary Fig.2 Age-stratified associations between sex hormones and CVD in male. Forest plots showing the associations between circulating sex hormones and risk of cardiovascular outcomes, stratified by study mean age (≥60 vs <60 years). (A) sex hormone–binding globulin (SHBG), (B) total testosterone (TT), (C) calculated free testosterone (cFT), (D) dihydrotestosterone (DHT), and (E) estradiol (E2). Squares represent hazard ratios (HRs) with 95% confidence intervals (CIs) for individual studies. The size of each square corresponds to the study's weight in the meta-analysis. Diamonds indicate the pooled estimates for each age subgroup (<60 years and ≥60
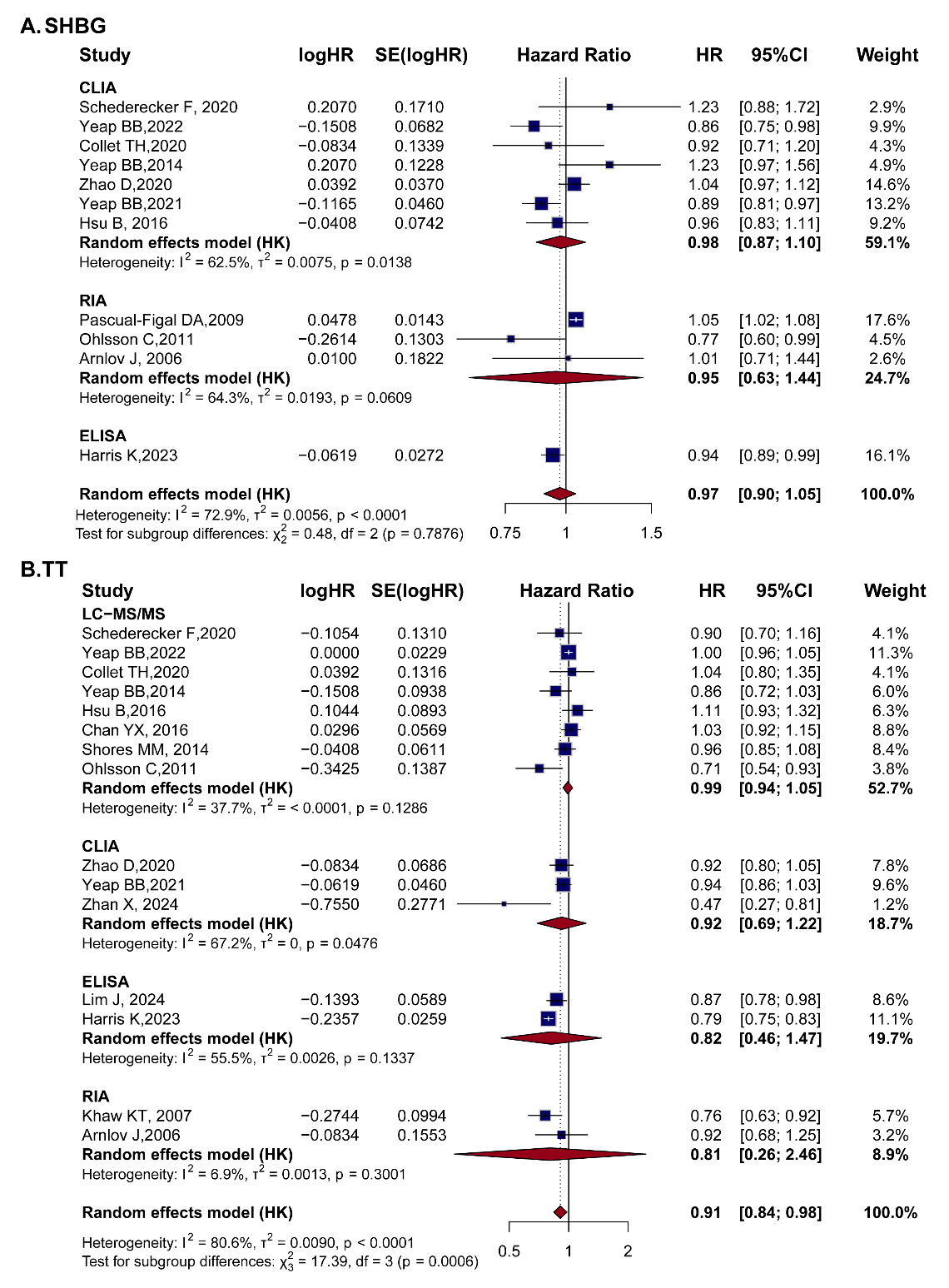
years).

Supplementary Fig.3 Forest plots showing the association between circulating sex hormones and the risk of CVD in men, stratified by assay technologies. (A) Sex hormone-binding globulin (SHBG), (B) Total testosterone (TT), and (C) calculated free testosterone (cFT). Squares represent hazard ratios (HRs) with 95% confidence intervals (CIs) for individual studies, with the size of each square proportional to the study’s weight in the meta-analysis. Diamonds indicate the pooled effect estimates for
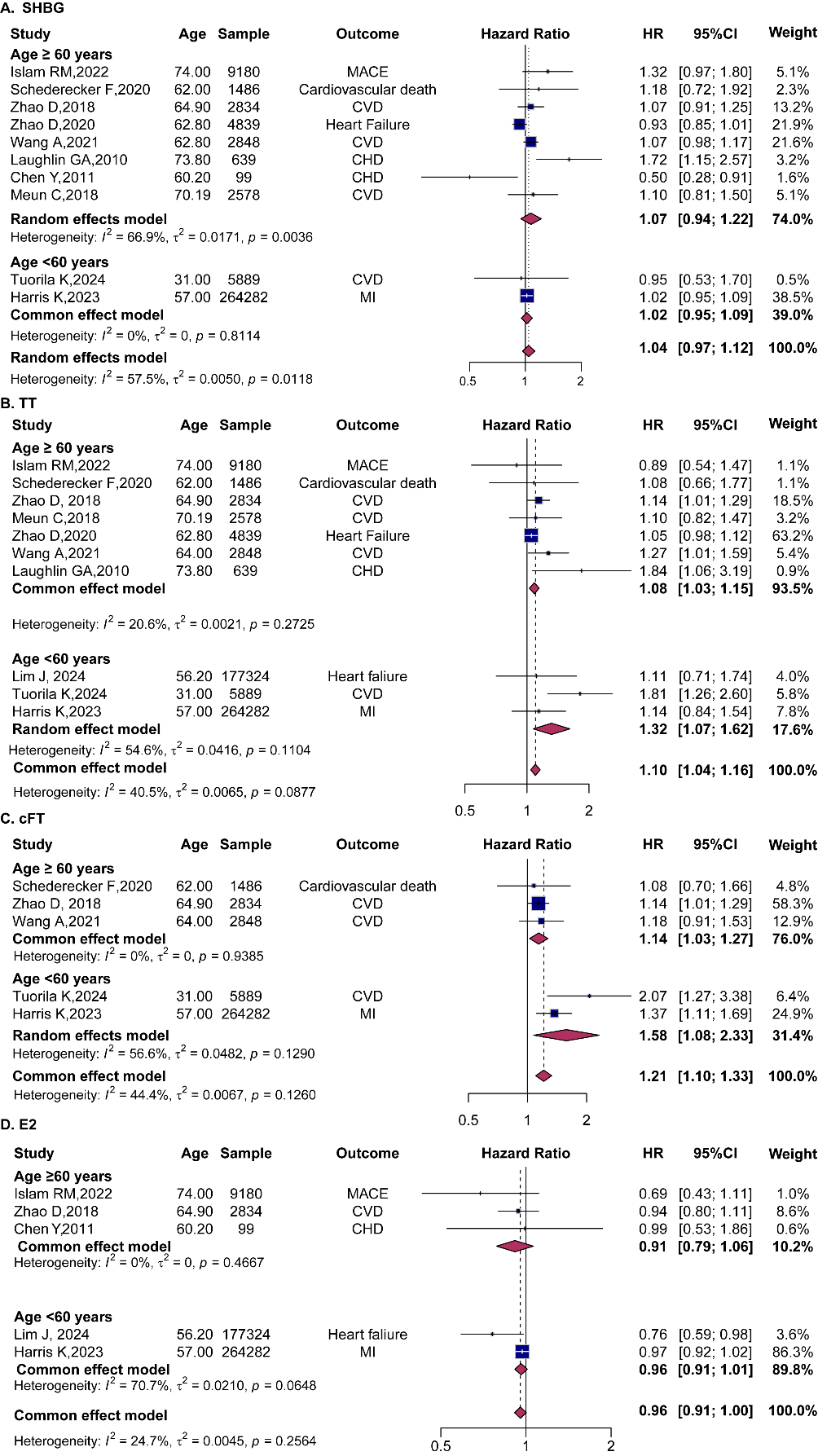
each assay subgroup (ELISA, RIA, CLIA, and LC–MS/MS).

Supplementary Fig.4 Age-stratified associations between sex hormones and CVD in female. Forest plots showing the associations between circulating sex hormones and risk of cardiovascular outcomes, stratified by study mean age (≥60 vs <60 years). (A) sex hormone–binding globulin (SHBG), (B) total testosterone (TT), (C) calculated free testosterone (cFT), (D) estradiol (E2). Squares represent hazard ratios (HRs) with 95% confidence intervals (CIs) for individual studies. The size of each square corresponds to the study's weight in the meta-analysis. Diamonds indicate the pooled estimates for each age subgroup (<60 years and ≥60 years).


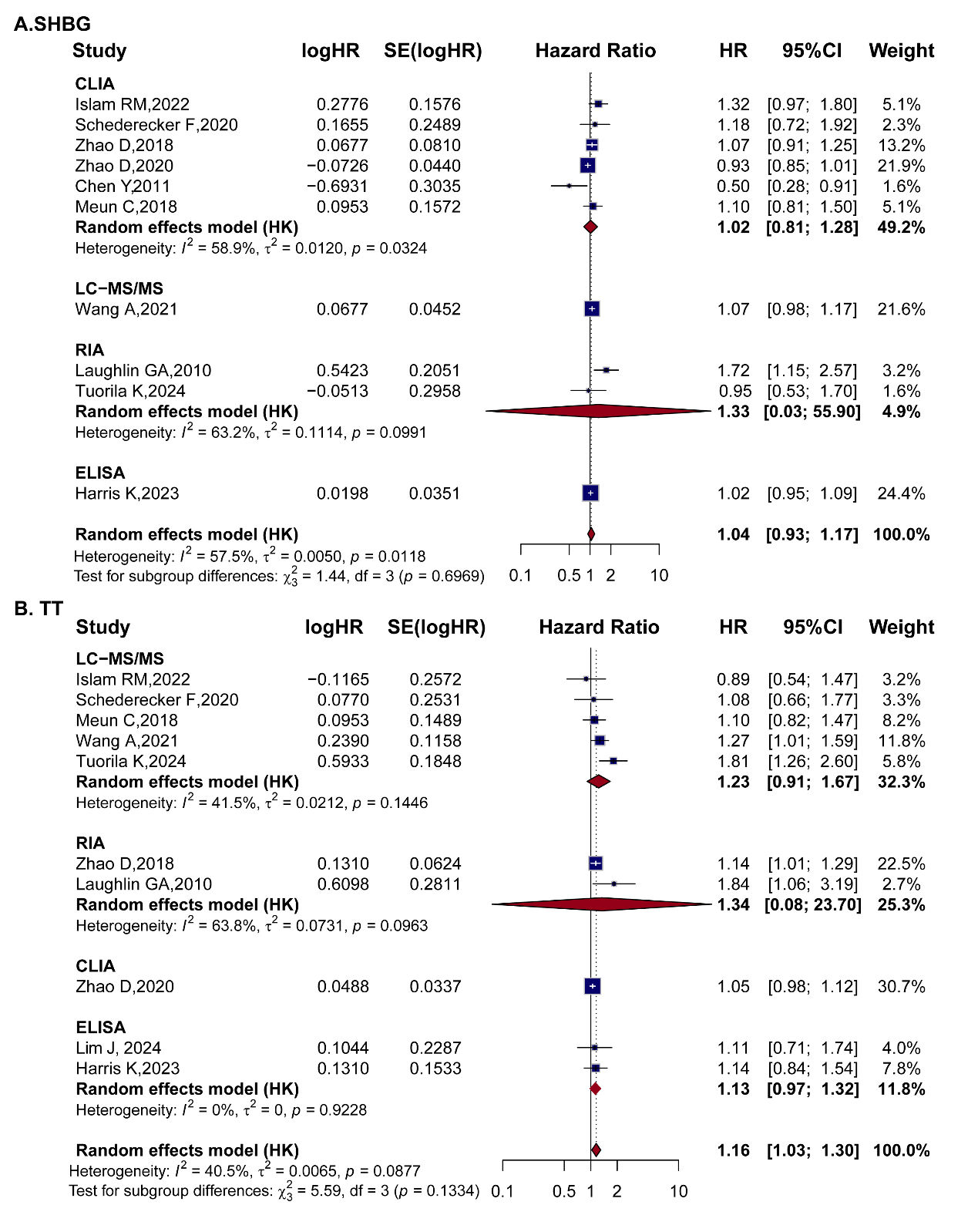
Supplementary Fig.5 Forest plots showing the association between circulating sex hormones and the risk of CVD in women, stratified by assay technologies. (A) Sex hormone-binding globulin (SHBG) and (B) Total testosterone (TT). Squares represent hazard ratios (HRs) with 95% confidence intervals (CIs) for individual studies, with square sizes proportional to the study’s weight in the meta-analysis. Diamonds indicate the pooled estimates for each assay subgroup (ELISA, RIA, CLIA, and LC–MS/MS).


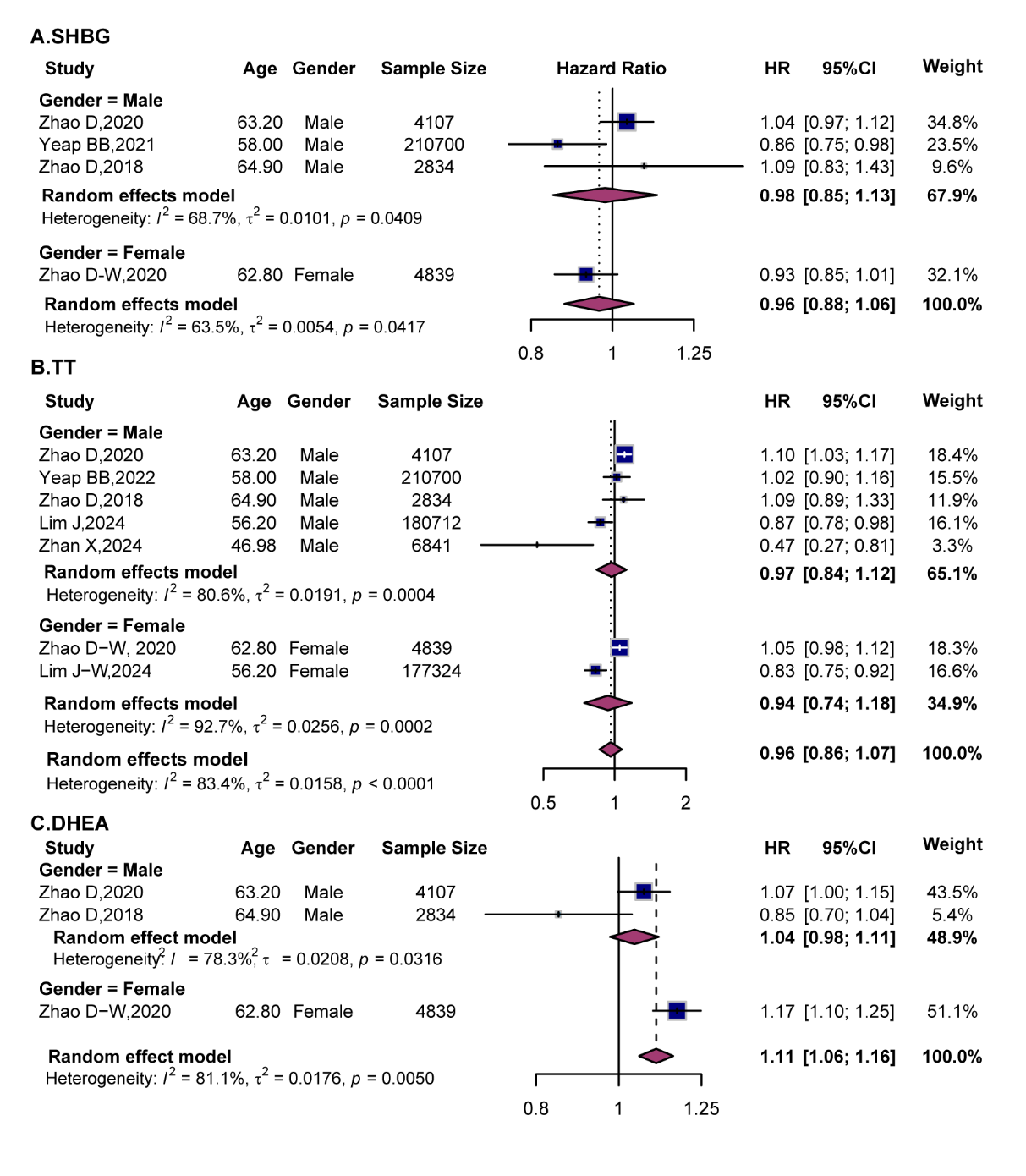
Supplementary Fig.6 Forest plots showing the association between sex hormones and the risk of heart failure, stratified by gender. (A) Sex hormone-binding globulin (SHBG), (B) Total testosterone (TT), and (C) Dehydroepiandrosterone (DHEA). Squares represent hazard ratios (HRs) with 95% confidence intervals (CIs) for individual studies. The size of each square corresponds to the study's weight in the meta-analysis. Diamonds indicate the pooled estimates for each gender subgroup (Male and Female).


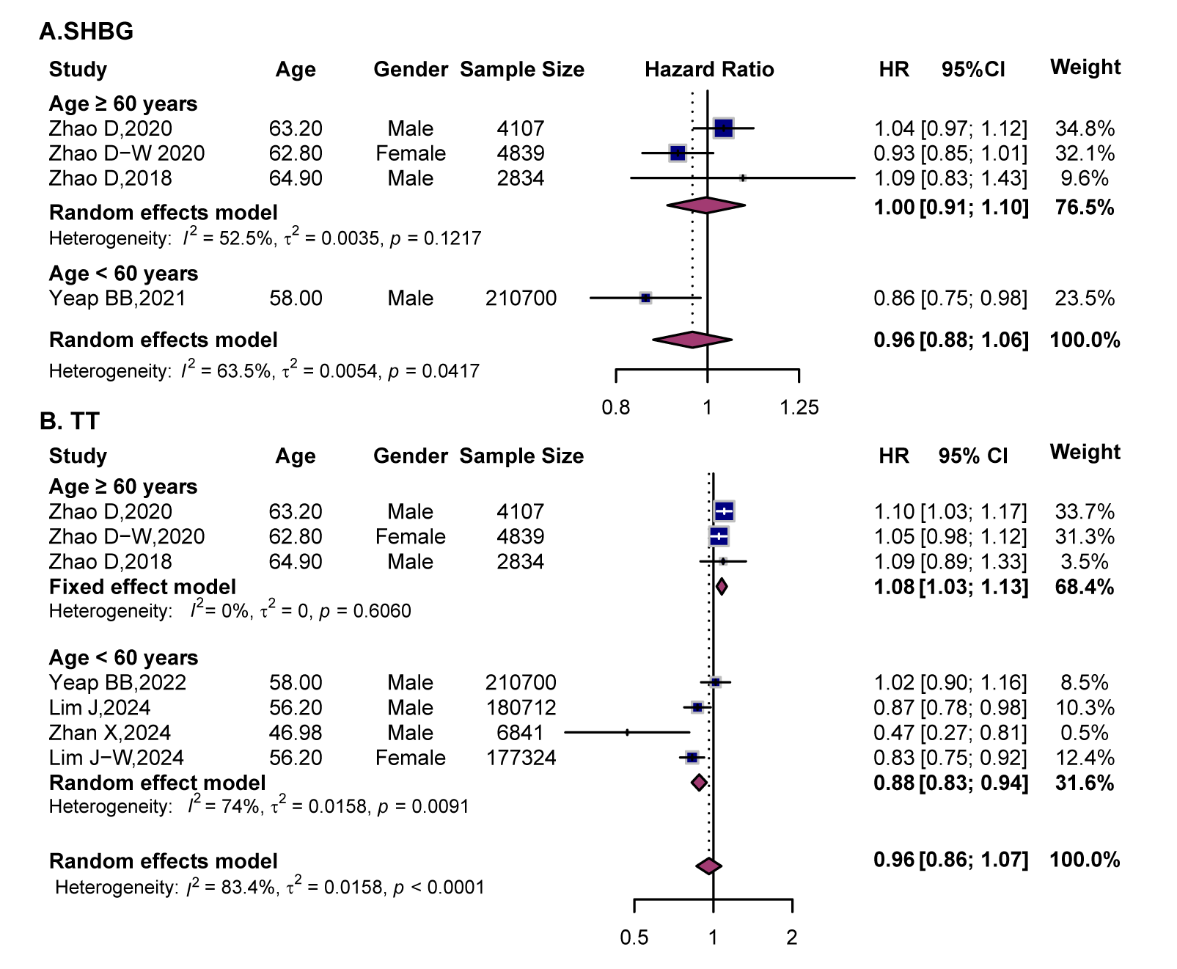
Supplementary Fig.7. Forest plots showing the association between sex hormones and the risk of heart failure, stratified by age. (A) Sex hormone-binding globulin (SHBG), (B) Total testosterone (TT). Squares represent hazard ratios (HRs) with 95% confidence intervals (CIs) for individual studies. The size of each square corresponds to the study's weight in the meta-analysis. Diamonds indicate the pooled estimates for each age subgroup (<60 years and ≥60 years).


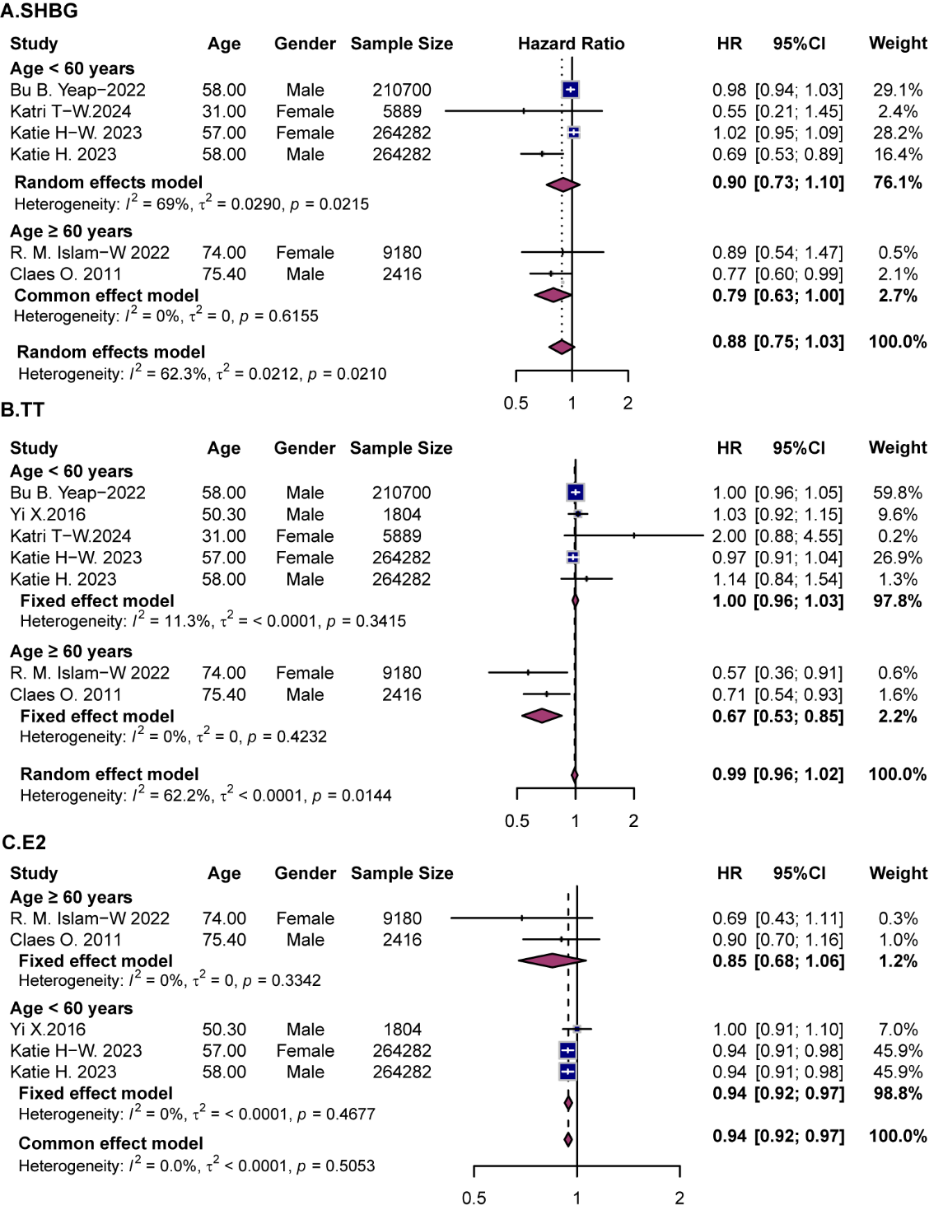

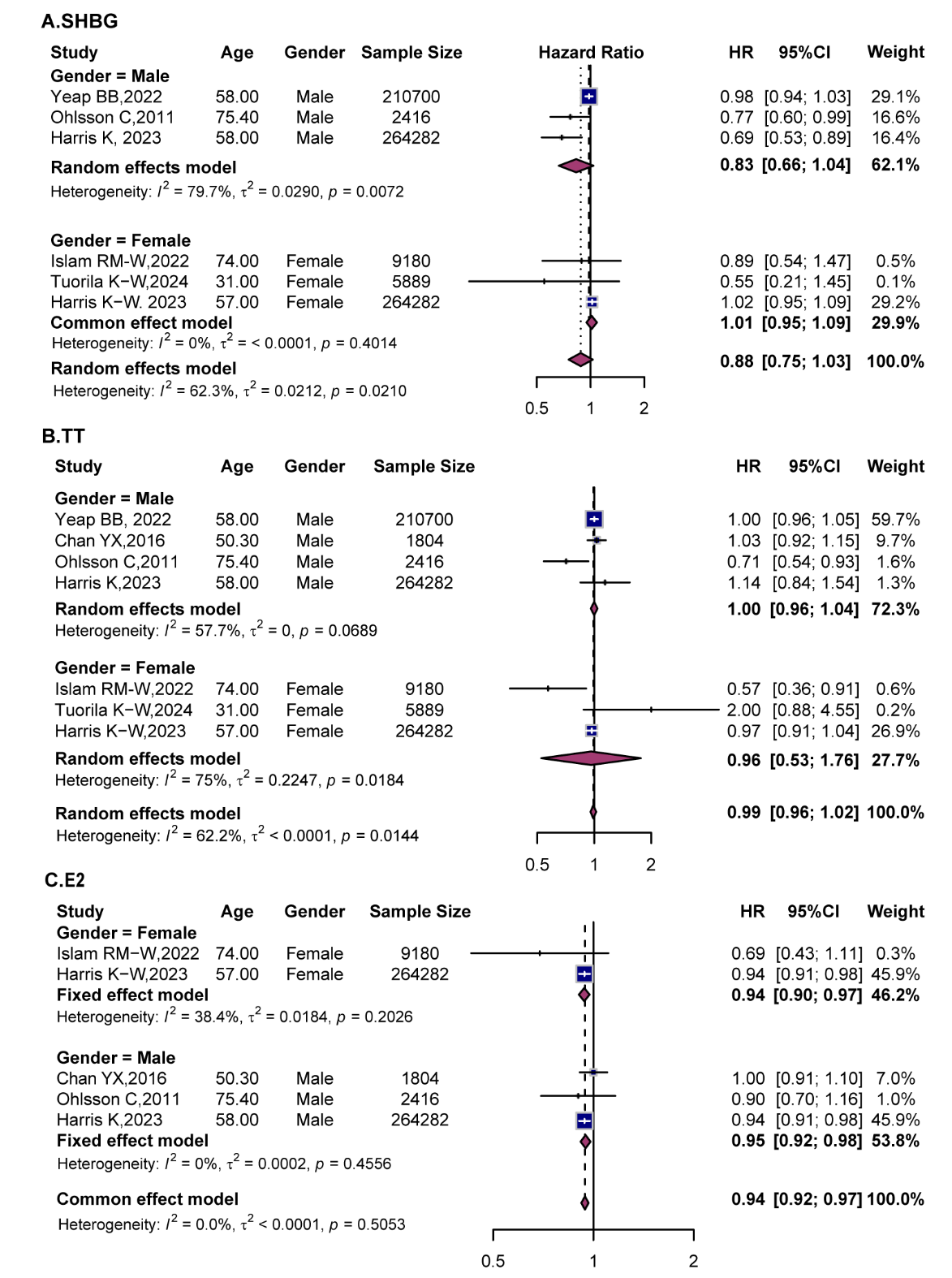
Supplementary Fig.8 Forest plots showing the association between sex hormones and the risk of major adverse cardiovascular events (MACE), stratified by gender. (A) Sex hormone-binding globulin (SHBG), (B) Total testosterone (TT), and (C) estradiol (E2). Squares represent hazard ratios (HRs) with 95% confidence intervals (CIs) for individual studies. The size of each square corresponds to the study's weight in the meta-analysis. Diamonds indicate the pooled estimates for each age subgroup (Male and Female).


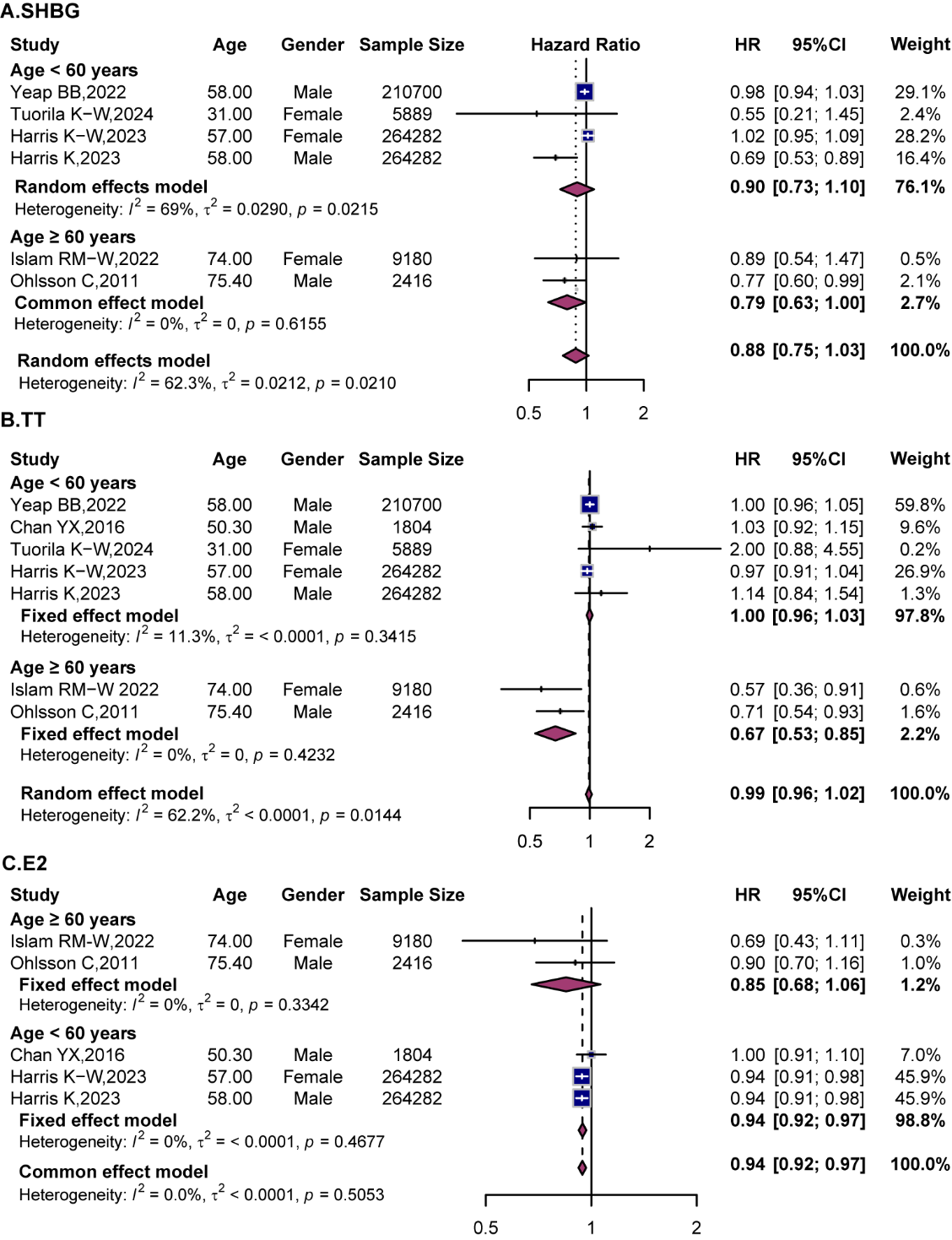
Supplementary Fig.9 Forest plots showing the association between sex hormones and the risk of major adverse cardiovascular events (MACE), stratified by age. (A) Sex hormone-binding globulin (SHBG), (B) Total testosterone (TT), and (C) estradiol (E2).. Squares represent hazard ratios (HRs) with 95% confidence intervals (CIs) for individual studies. The size of each square corresponds to the study's weight in the meta-analysis. Diamonds indicate the pooled estimates for each age subgroup (<60 years and ≥60 years).


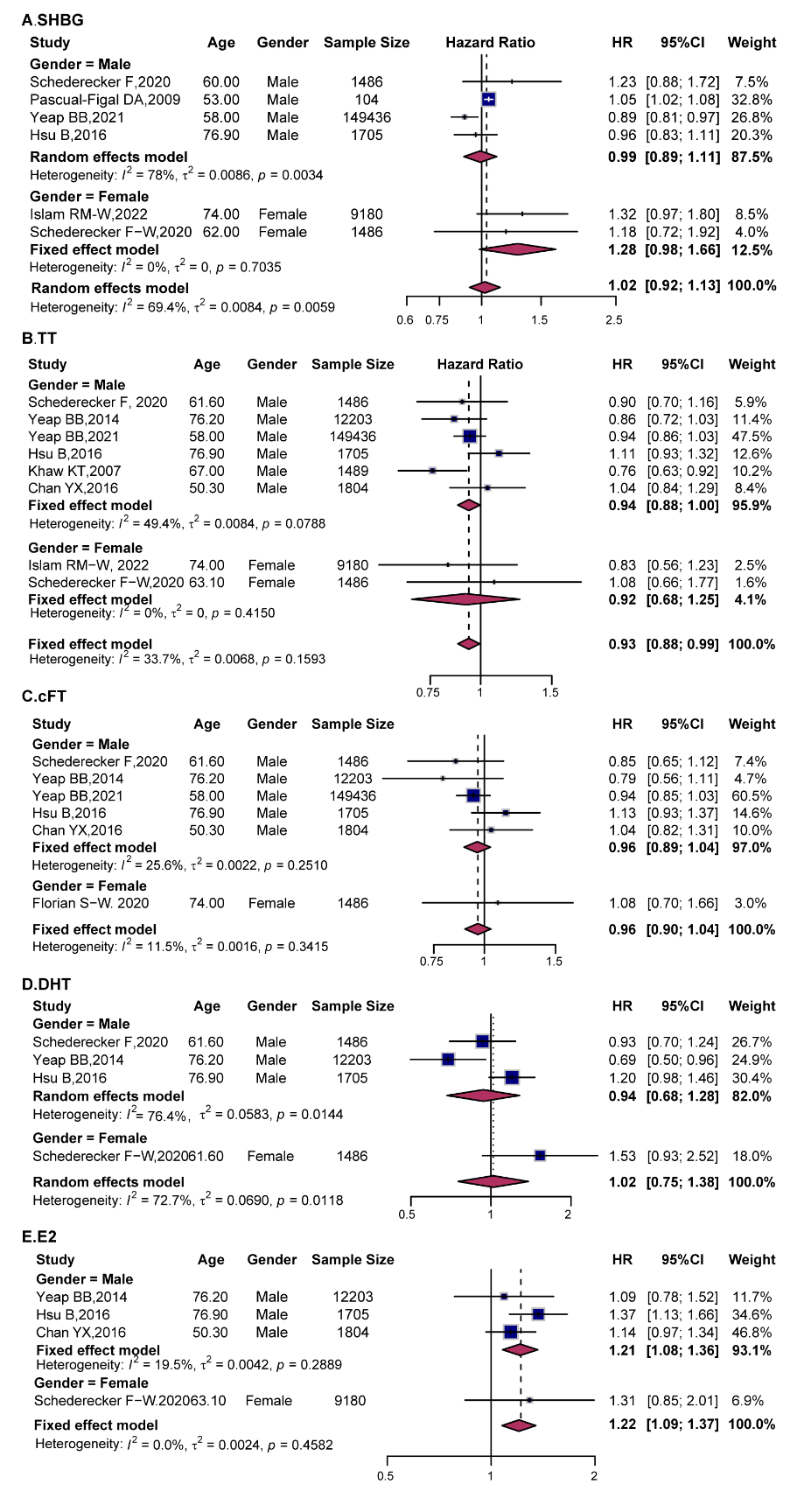
Supplementary Fig.10 Forest plots showing the association between sex hormones and the risk of cardiovascular mortality, stratified by gender. (A) Total testosterone (TT), (B) Sex hormone-binding globulin (SHBG), (C) Calculated free testosterone (cFT), and (D) Dihydrotestosterone (DHT), (E) estradiol (E2). Squares represent hazard ratios (HRs) with 95% confidence intervals (CIs) for individual studies. The size of each square corresponds to the study's weight in the meta-analysis. Diamonds indicate the pooled estimates for each age subgroup (Male and Female).


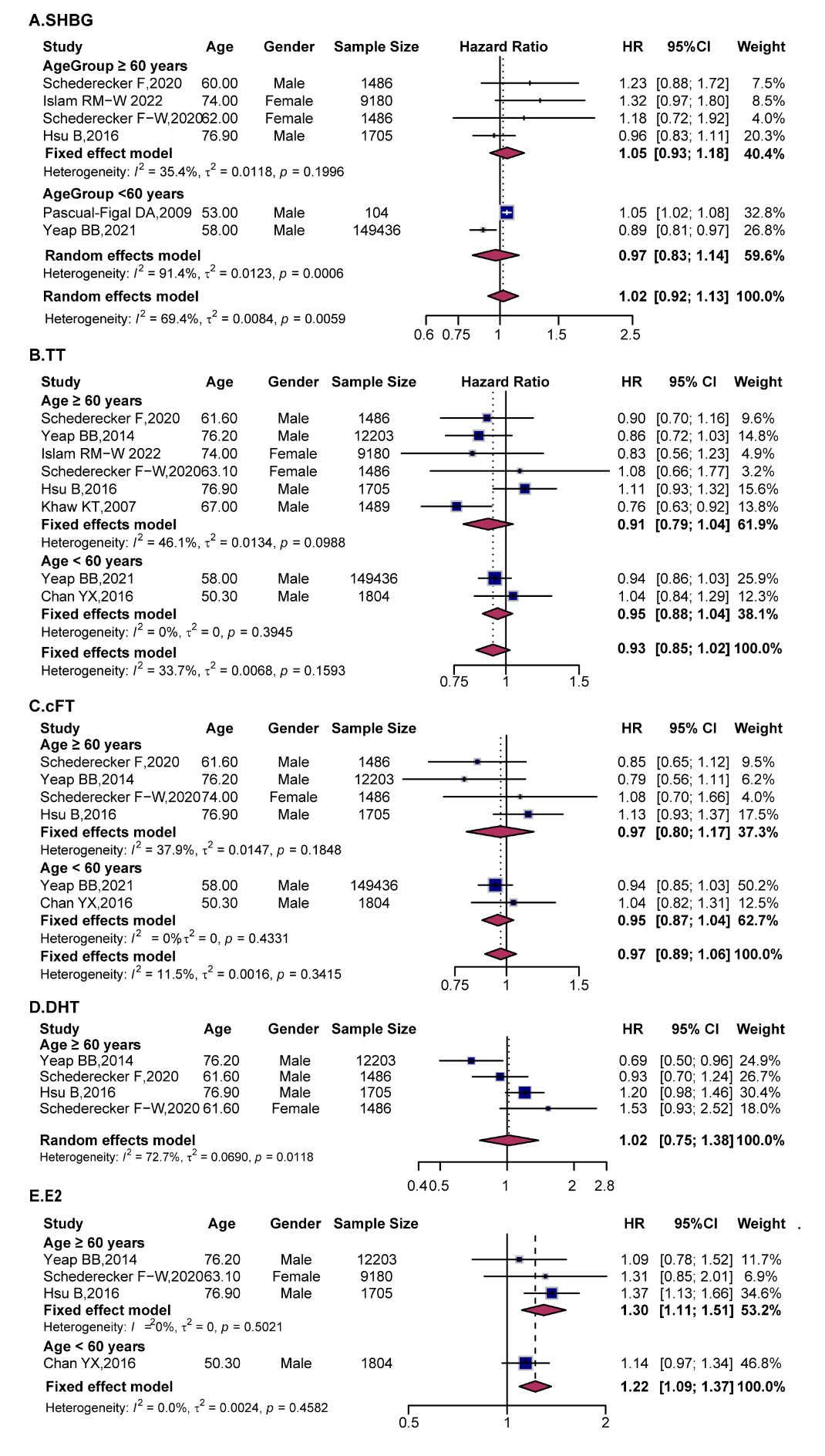
Supplementary Fig.11 Forest plots showing the association between sex hormones and the risk of cardiovascular mortality, stratified by age. (A) Total testosterone (TT), (B) Sex hormone-binding globulin (SHBG), (C) Calculated free testosterone (cFT), and (D) Dihydrotestosterone (DHT), (E) estradiol (E2). Squares represent hazard ratios (HRs) with 95% confidence intervals (CIs) for individual studies. The size of each square corresponds to the study's weight in the meta-analysis. Diamonds indicate the pooled estimates for each age subgroup (<60 years and ≥60 years).


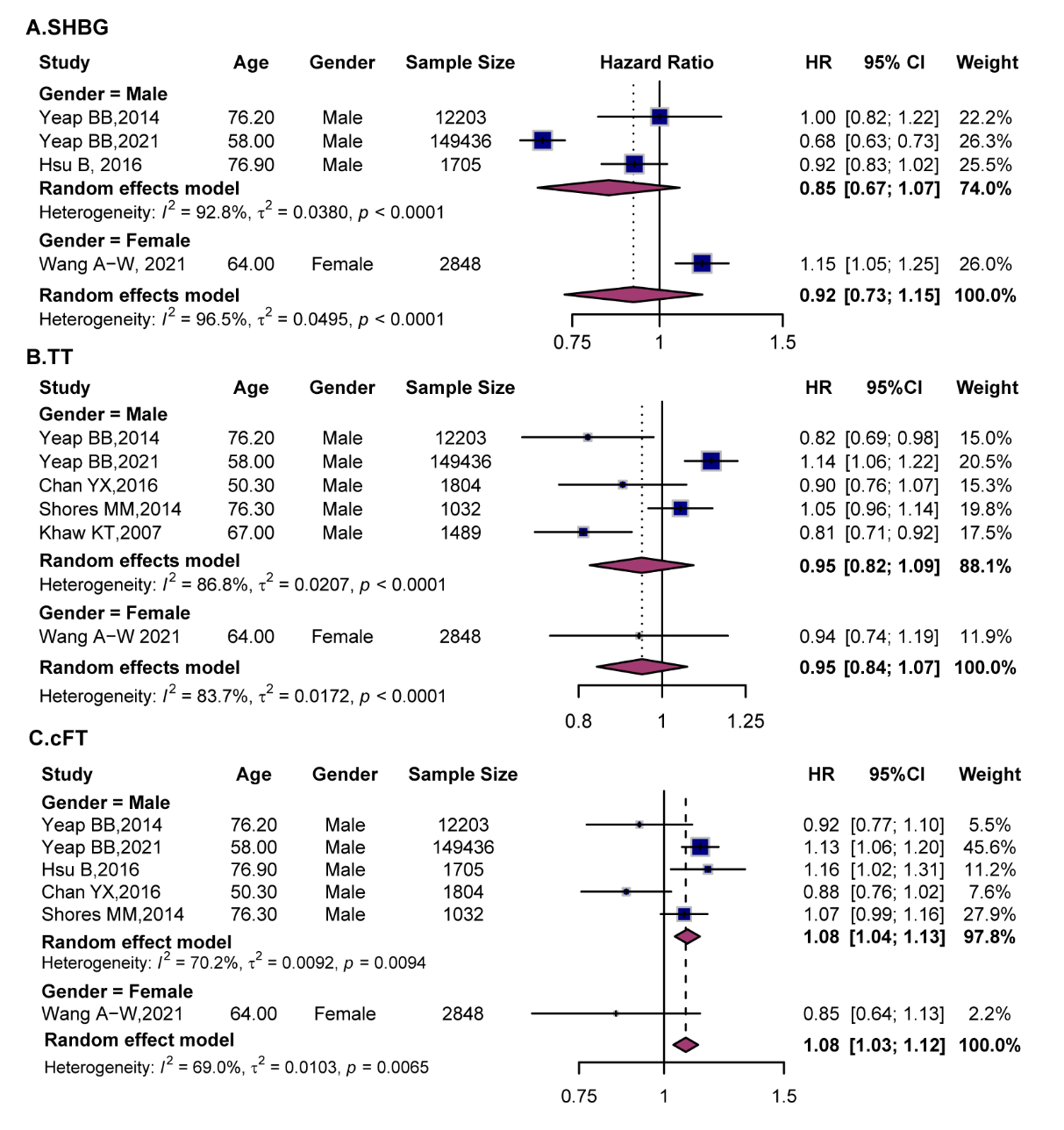
Supplementary Fig.12 Forest plots showing the association between sex hormones and the risk of all-cause mortality, stratified by gender. (A) Sex hormone-binding globulin (SHBG), (B) Total testosterone (TT), (C) calculated free testosterone (cFT) Squares represent hazard ratios (HRs) with 95% confidence intervals (CIs) for individual studies. The size of each square corresponds to the study's weight in the meta-analysis. Diamonds indicate the pooled estimates for each age subgroup (Male and Female).


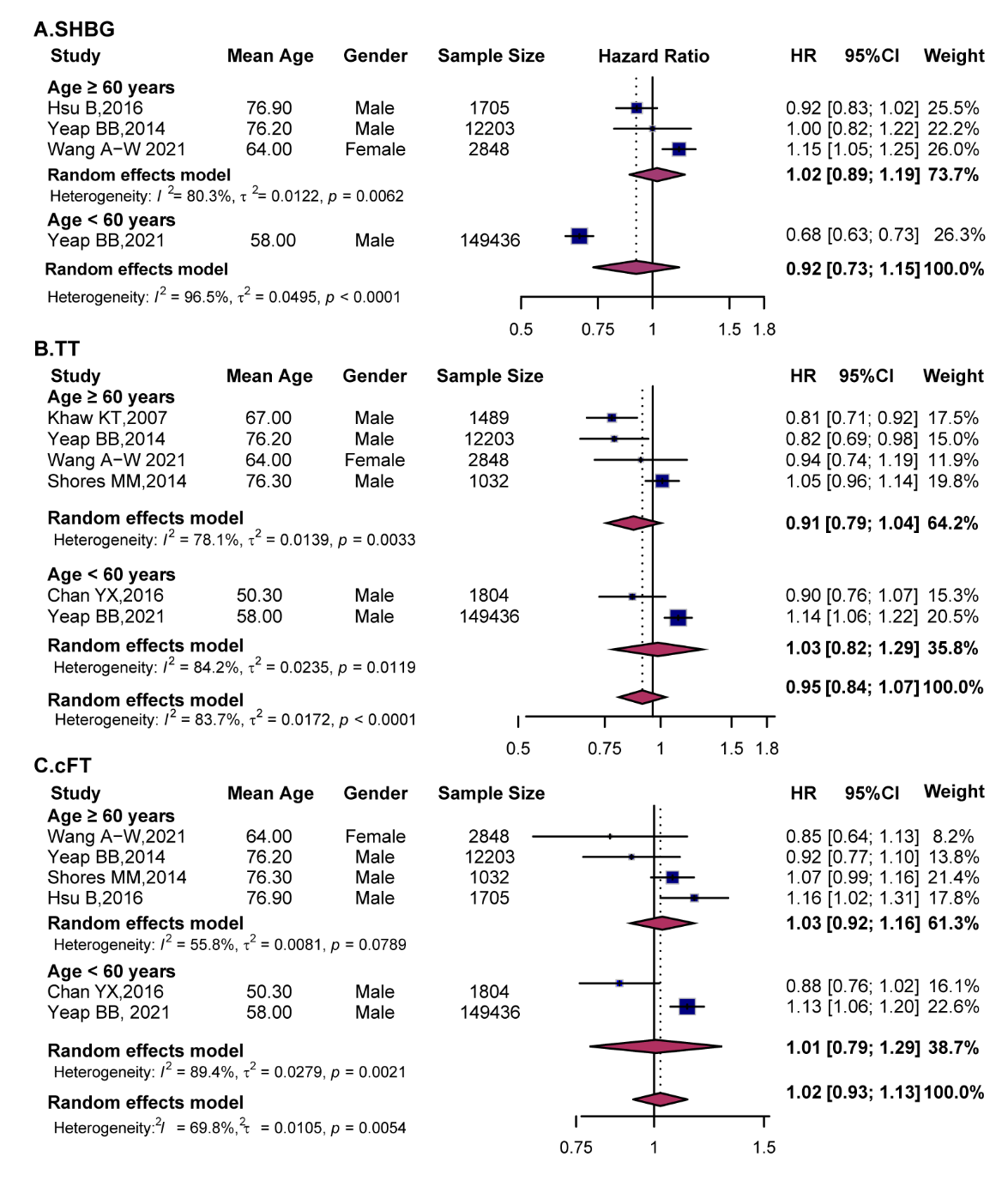
Supplementary Fig.13 Forest plots showing the association between sex hormones and the risk of all-cause mortality, stratified by age. (A) Sex hormone-binding globulin (SHBG), (B) Total testosterone (TT), (C) calculated free testosterone (cFT) Squares represent hazard ratios (HRs) with 95% confidence intervals (CIs) for individual studies. The size of each square corresponds to the study's weight in the meta-analysis. Diamonds indicate the pooled estimates for each age subgroup (<60 years and ≥60 years).


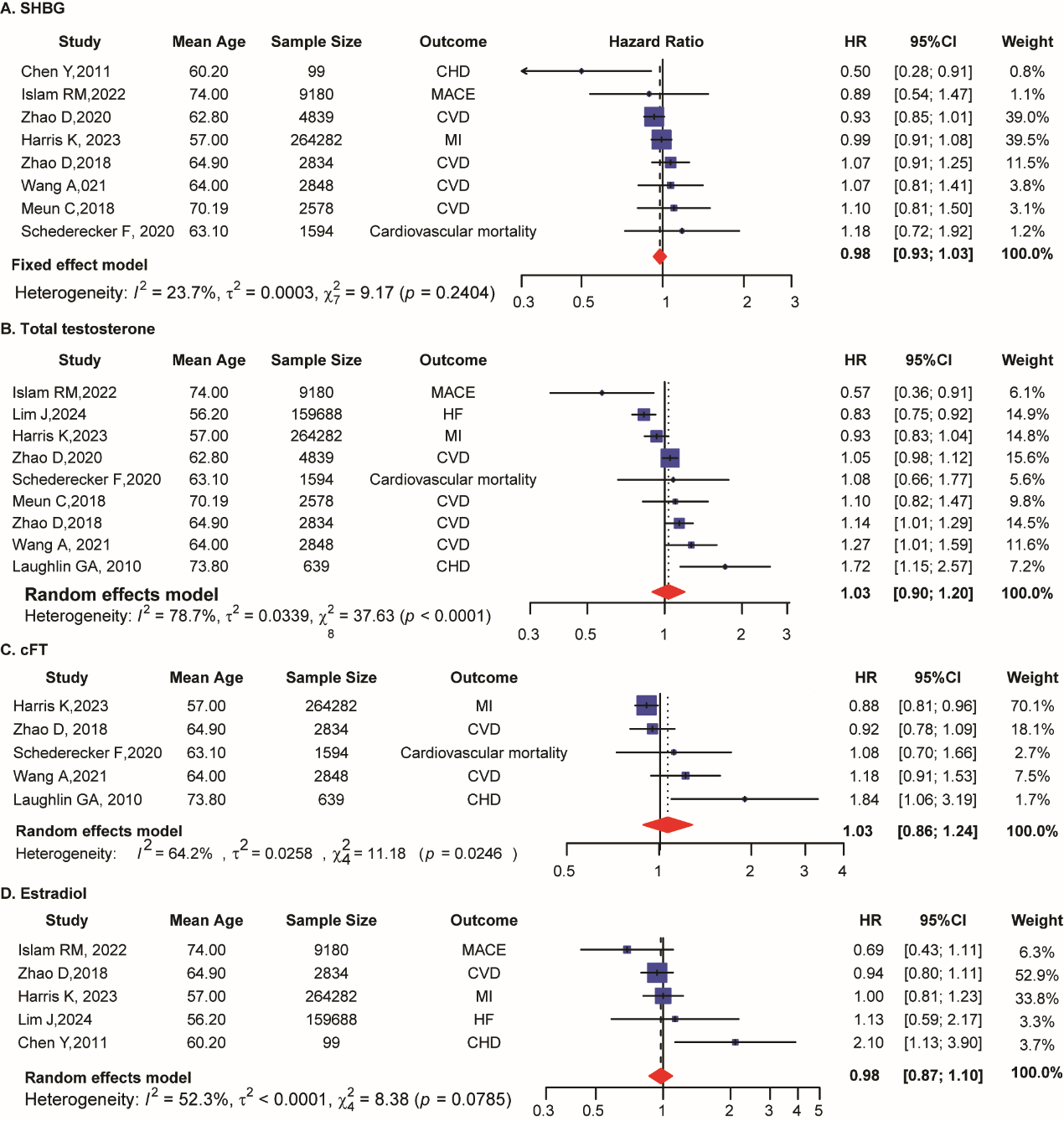
Supplementary Fig.14 Forest plots showing the association between sex hormones and the risk of postmenopausal women. (A) Sex hormone-binding globulin (SHBG), (B) Total testosterone, (C) Calculated free testosterone (cFT) and (D) Estradiol. Squares represent hazard ratios (HRs) with 95% confidence intervals (CIs) for individual studies.


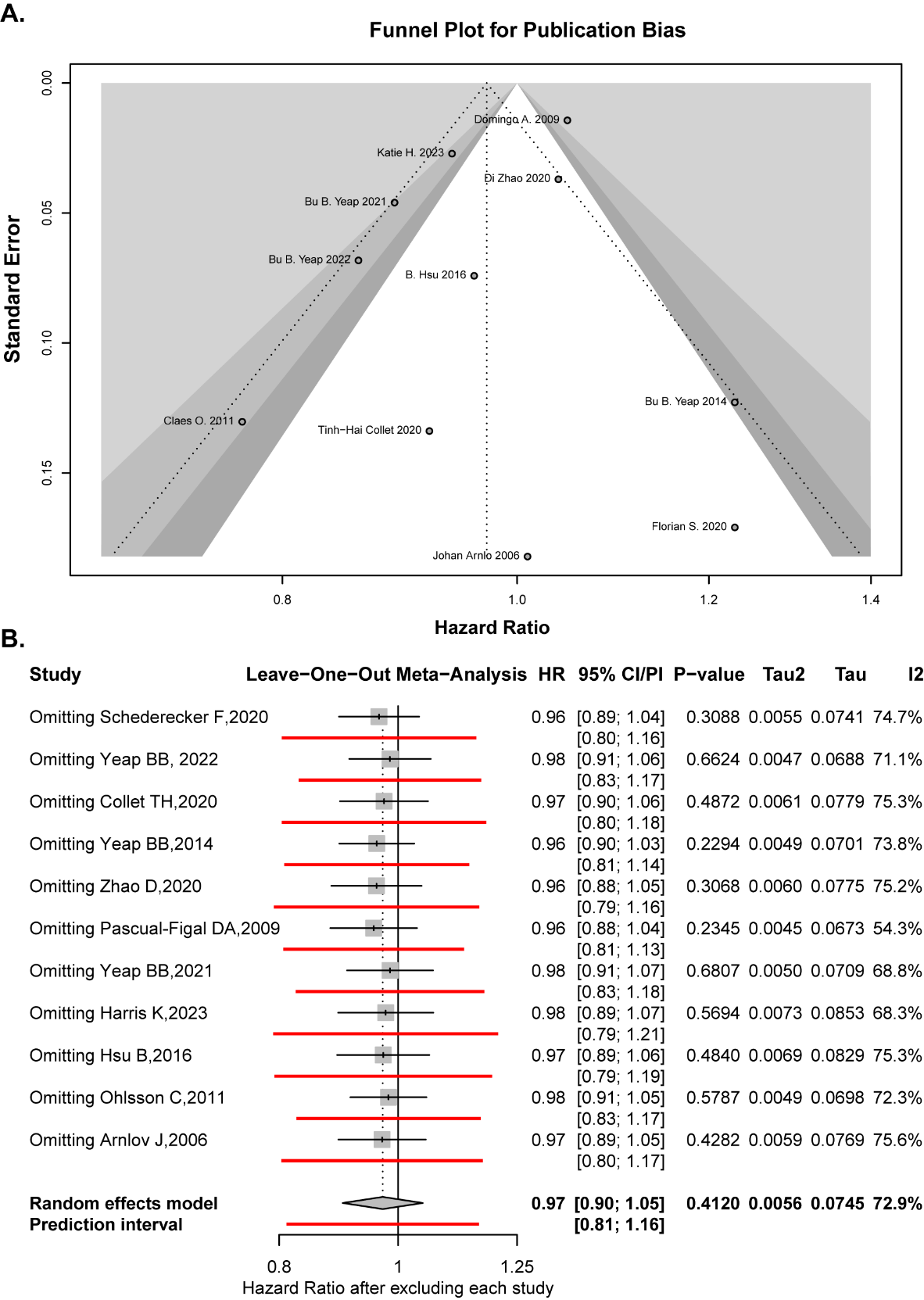
Supplementary Fig.15 Funnel plot and leave-one-out sensitivity analysis for the association between SHBG and CVD risk in men. (A) Funnel plot displaying study-specific hazard ratios (HRs) plotted against their standard errors, used to evaluate potential publication bias. The degree of symmetry reflects the likelihood of small-study effects. (B) Leave-one-out sensitivity analysis, where each study is sequentially omitted to assess the stability of the pooled effect estimate. Squares represent recalculated pooled HRs after exclusion of each individual study, and horizontal lines denote the corresponding 95% confidence intervals. Consistent findings across iterations indicate strong robustness of the meta-analytic results.


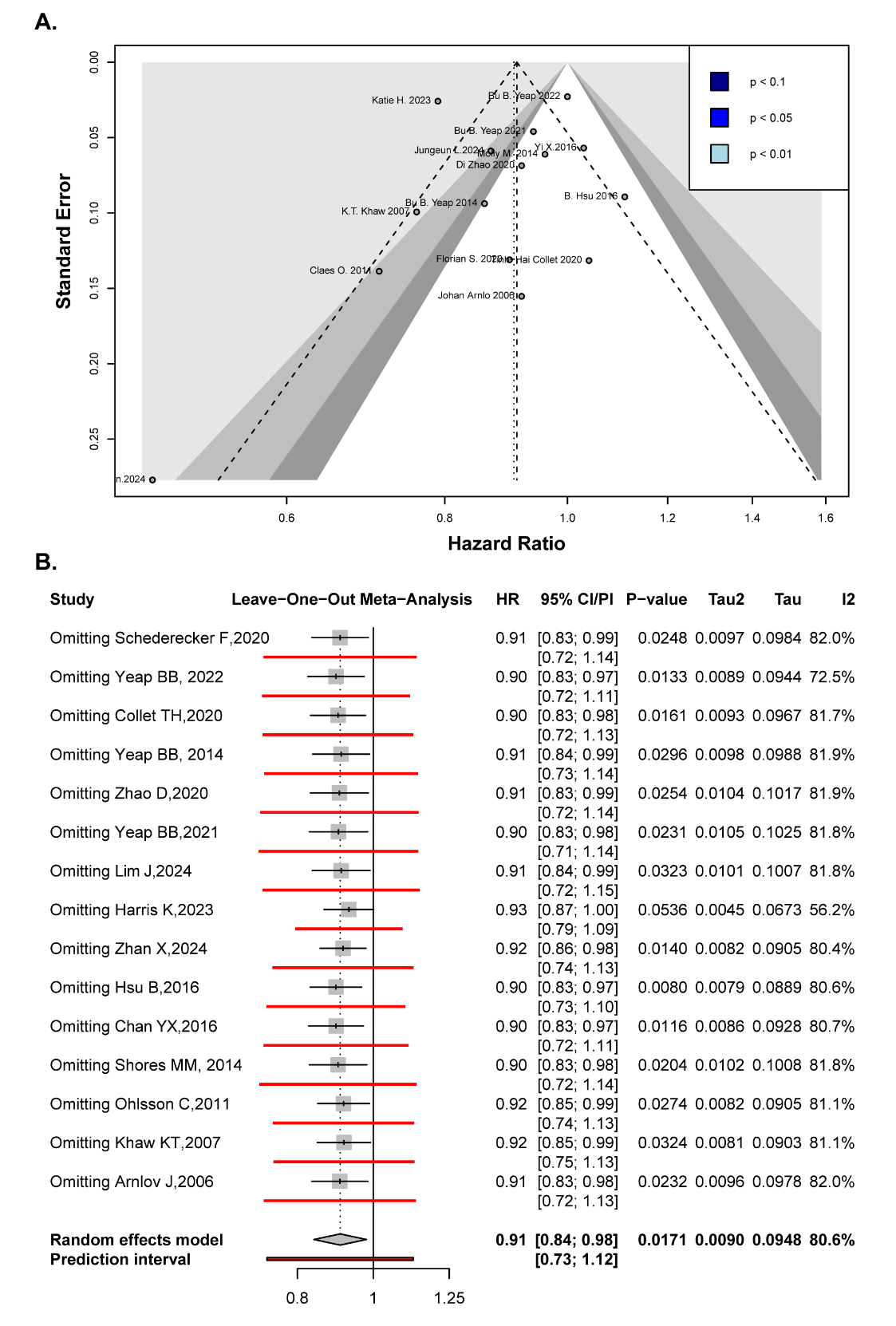
Supplementary Fig.16 Funnel plot and leave-one-out sensitivity analysis for the association between TT and CVD risk in men. (A) Funnel plot depicting study-specific hazard ratios (HRs) plotted against their standard errors to assess the presence of small-study effects. Symmetry of the plot suggests a lower likelihood of publication bias. (B) Leave-one-out sensitivity analysis, in which each study is sequentially omitted to examine the stability of the pooled estimate. Squares represent pooled HRs recalculated after omitting each individual study, and horizontal lines indicate the corresponding 95% confidence intervals. Consistent effect estimates across exclusions support the robustness of the meta-analytic findings.


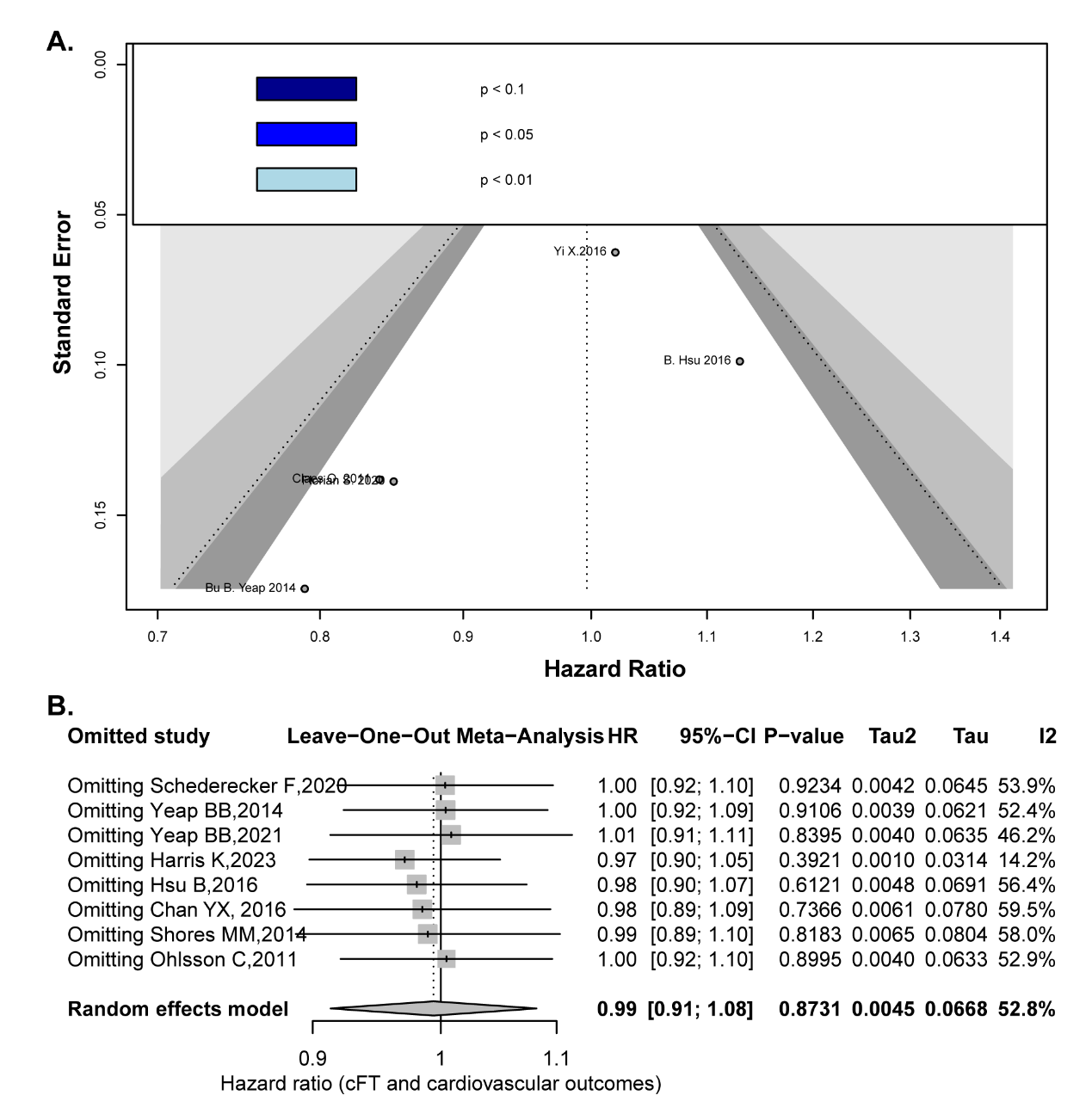
Supplementary Fig.17 Funnel plot and leave-one-out sensitivity analysis for the association between cFT and CVD risk in men. (A) Funnel plot evaluating potential publication bias, with hazard ratios (HRs) plotted against their standard errors. Symmetry of the plot reflects low likelihood of small-study effects. (B) Leave-one-out sensitivity analysis, in which each study is sequentially omitted to evaluate the stability of the pooled effect estimate. Squares represent the pooled HRs after omitting each individual study, with horizontal lines indicating the corresponding 95% confidence intervals. Consistent effect estimates across omissions indicate good robustness of the meta-analysis results.


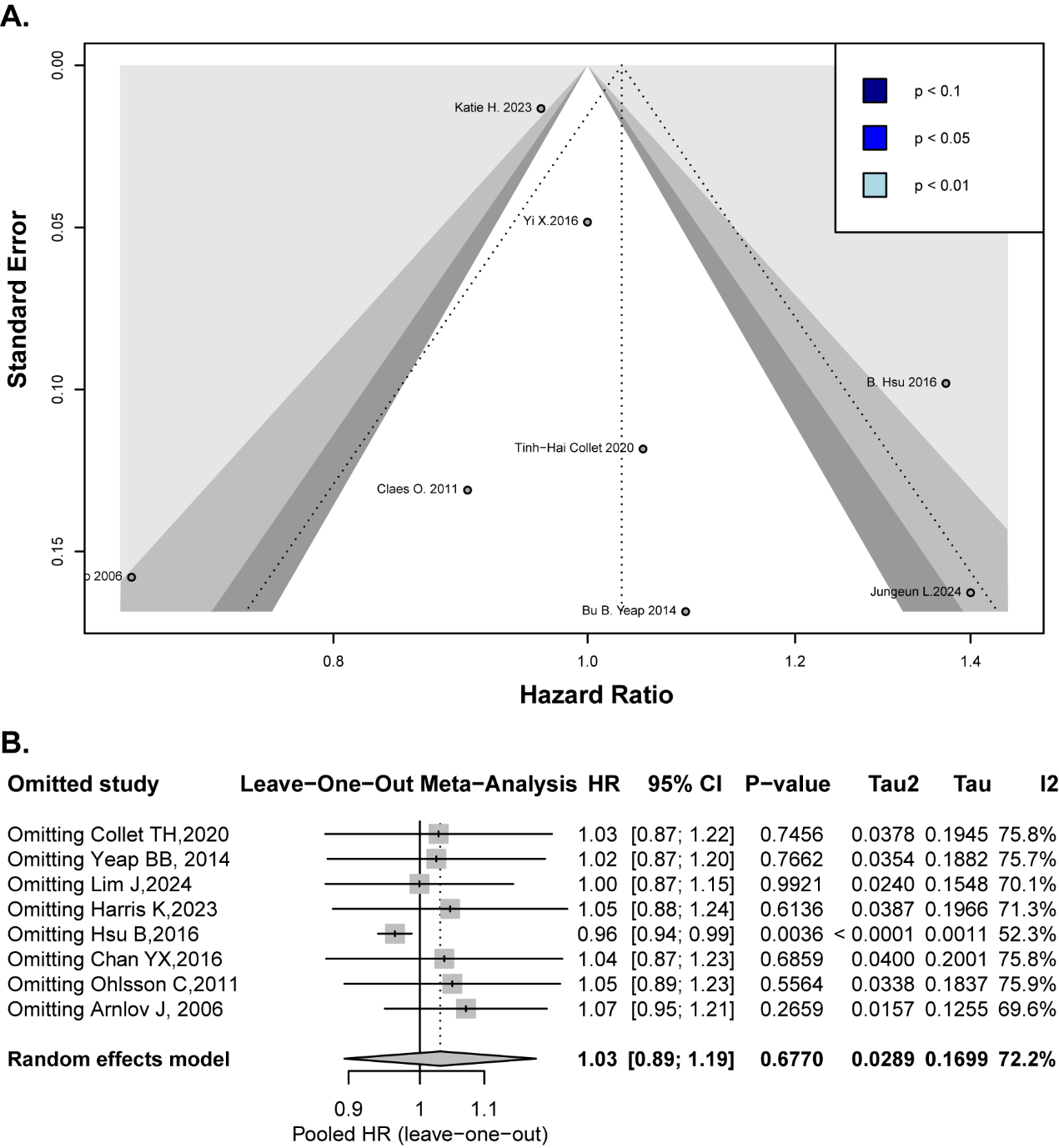
Supplementary Fig.18 Funnel plot and leave-one-out sensitivity analysis for the association between E2 and CVD risk in men. (A) Funnel plot showing the distribution of study-specific hazard ratios (HRs) against their standard errors. The degree of symmetry reflects the presence or absence of small-study effects. (B) Leave-one-out sensitivity analysis, in which each study is sequentially omitted to evaluate the stability of the pooled effect estimate. Squares represent the pooled HRs after omitting each individual study, with horizontal lines indicating the corresponding 95% confidence intervals. Consistent effect estimates across omissions indicate good robustness of the meta-analysis results.


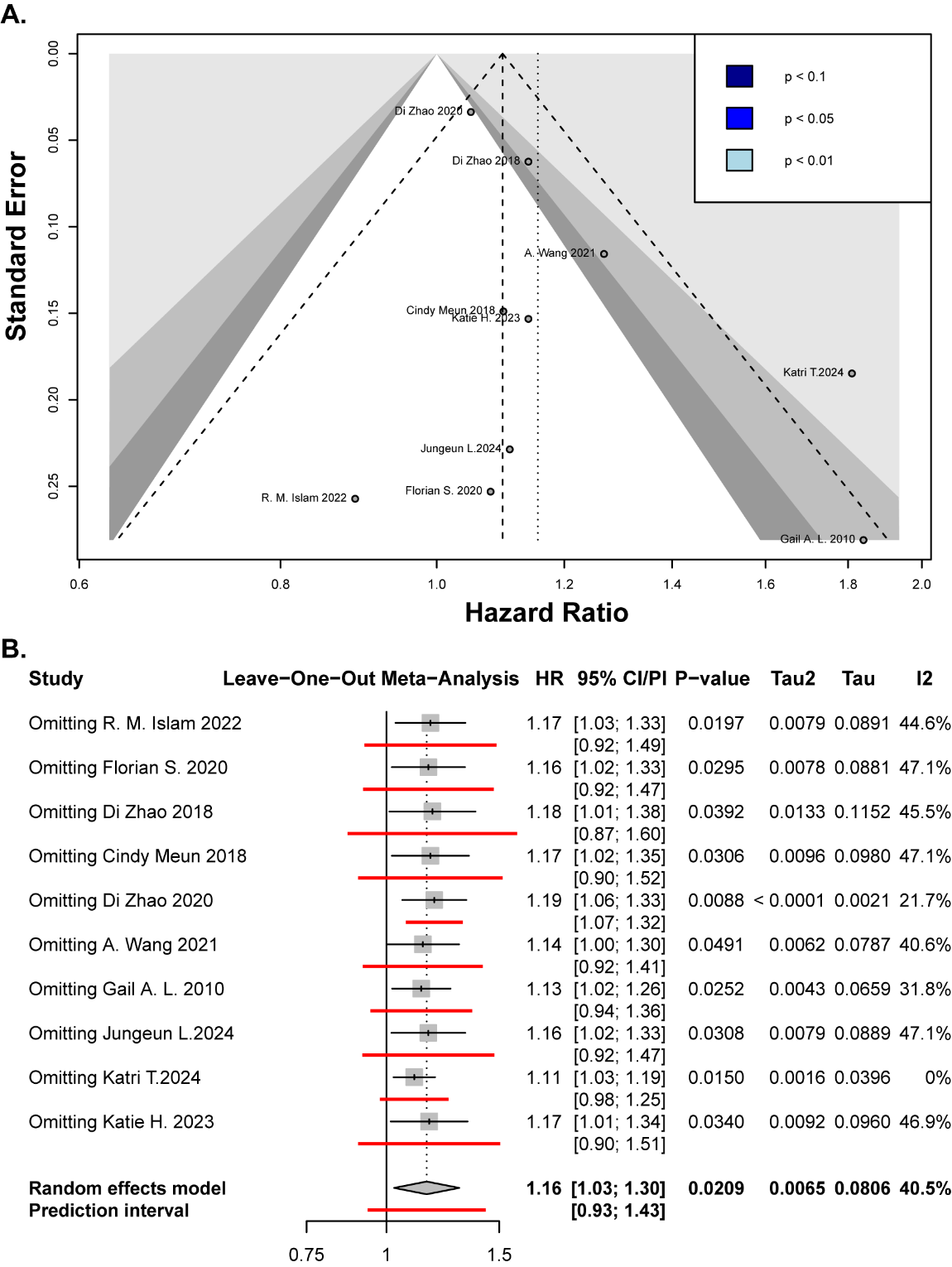

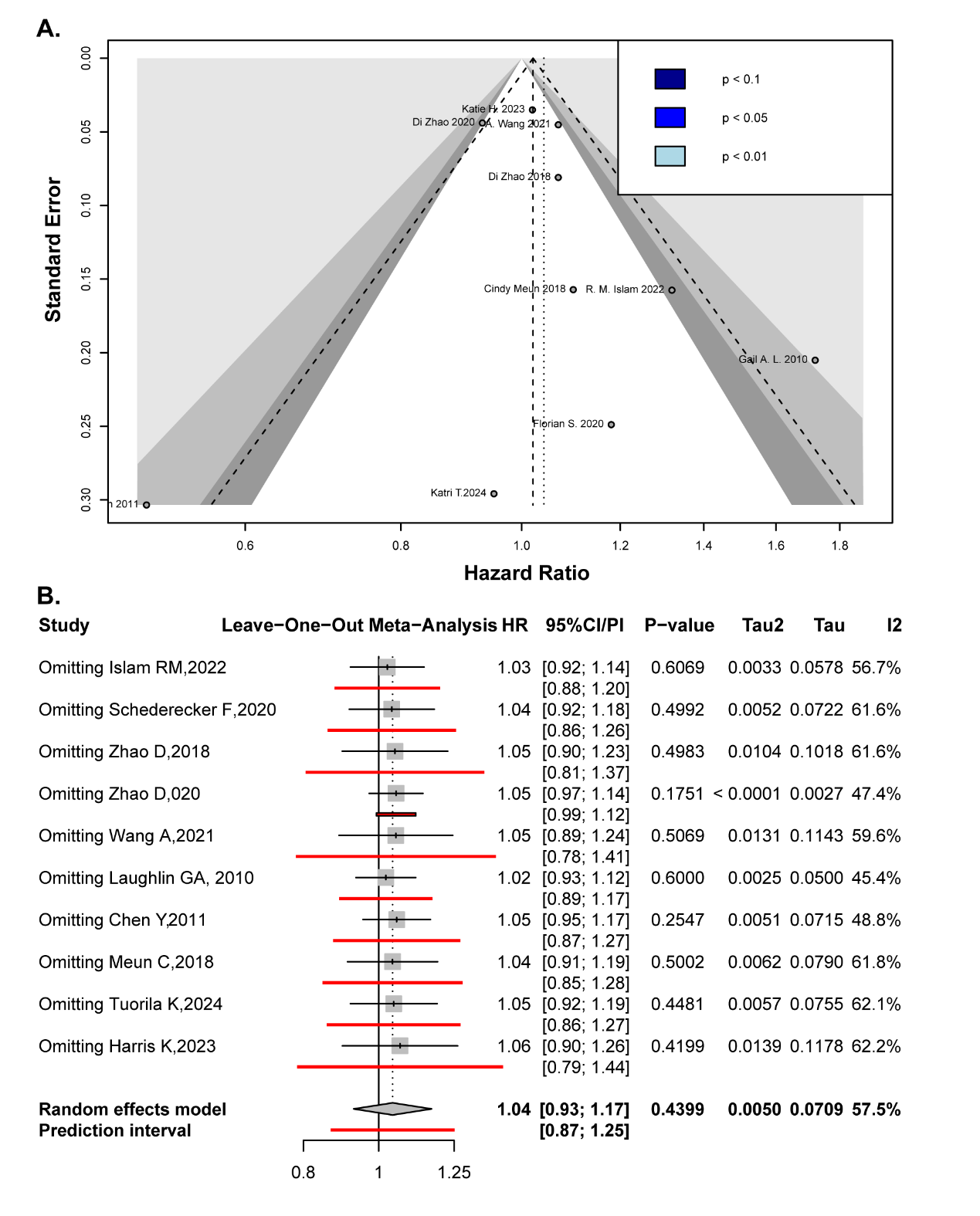
Supplementary Fig.19 Funnel plot and leave-one-out sensitivity analysis for the association between SHBG and CVD risk in female. (A) Funnel plot showing study-specific hazard ratios (HRs) plotted against their standard errors to assess potential small-study effects. Greater symmetry in the plot suggests a lower likelihood of publication bias. (B) Leave-one-out sensitivity analysis in which each study is sequentially excluded to evaluate the stability of the pooled effect estimate. Squares represent recalculated pooled HRs after omitting each study, and horizontal lines denote the corresponding 95% confidence intervals. Consistent results across exclusions indicate the robustness of the meta-analysis findings.


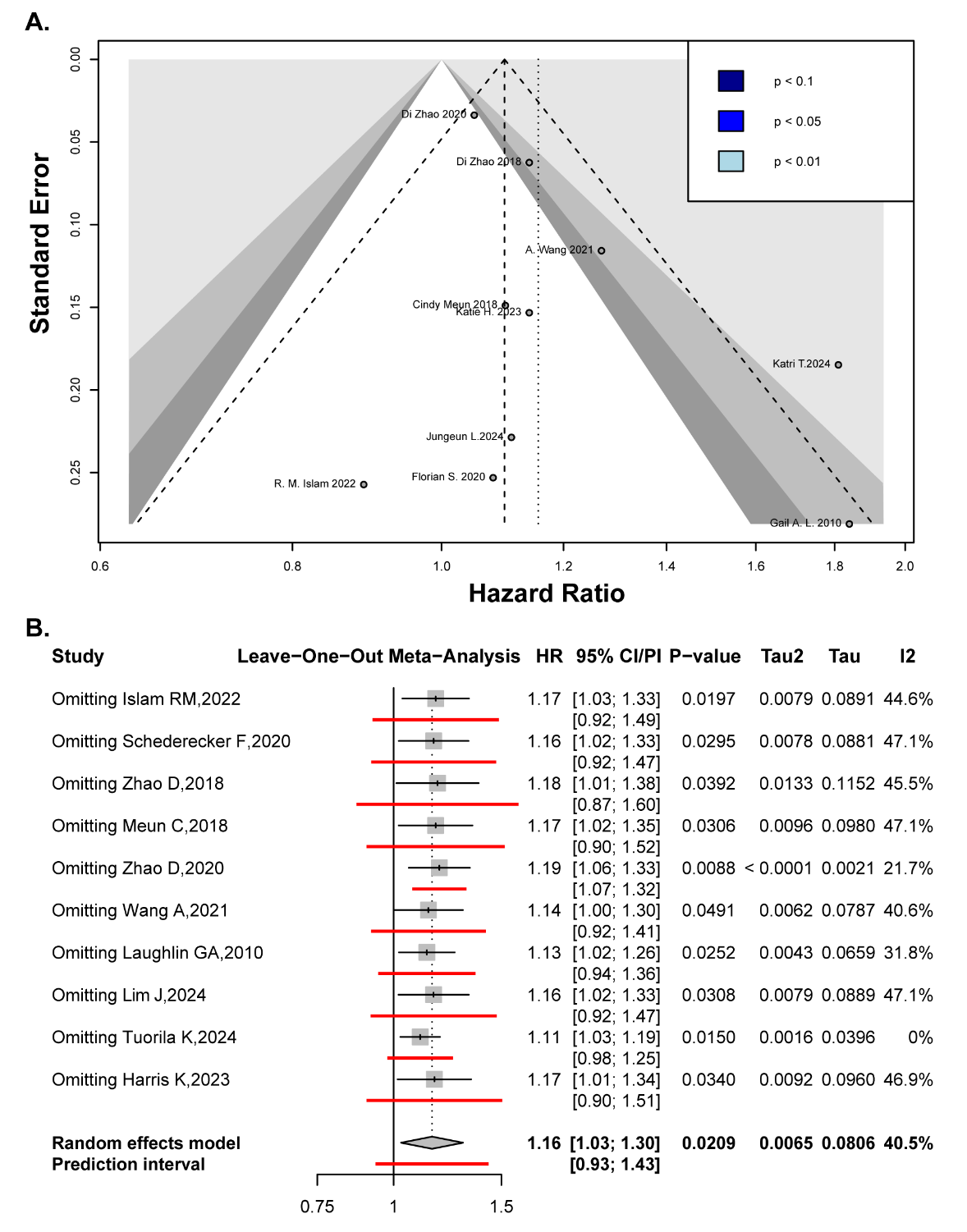
Supplementary Fig.20 Funnel plot and leave-one-out sensitivity analysis for the association between TT and CVD risk in female. (A) Funnel plot presenting study-specific hazard ratios (HRs) versus their standard errors, used to evaluate potential small-study effects. Symmetry of the funnel plot suggests a lower likelihood of publication bias. (B) Leave-one-out sensitivity analysis, in which each included study is sequentially omitted to examine the stability of the pooled effect estimate. Squares represent recalculated pooled HRs following the exclusion of each individual study, with horizontal lines indicating corresponding 95% confidence intervals. Stable estimates across iterations support the robustness of the meta-analytic findings.


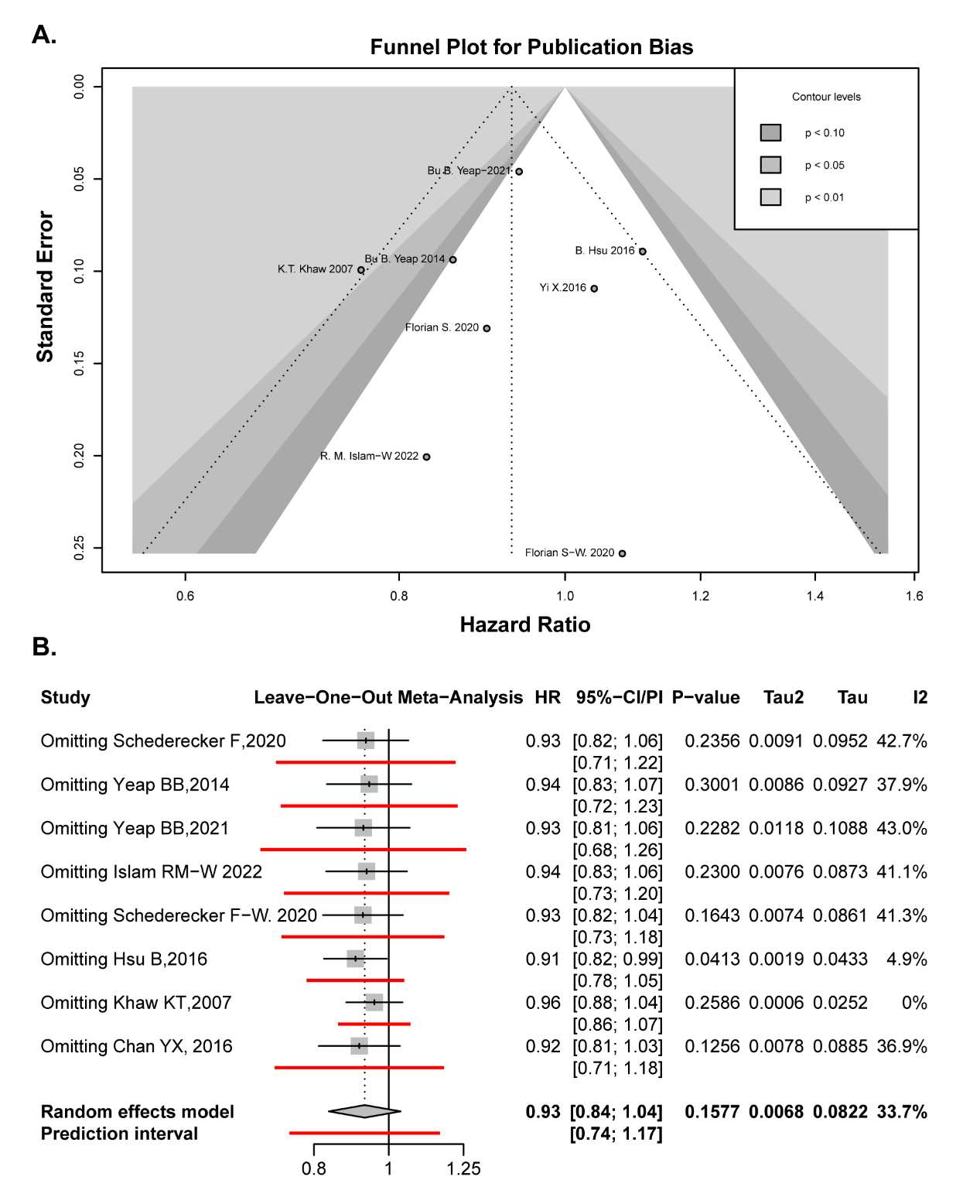
Supplementary Fig.21 Funnel plot and leave-one-out sensitivity analysis for the association between TT and cardiovascular mortality. (A) Funnel plot showing study-specific hazard ratios (HRs) plotted against their standard errors, used to evaluate potential publication bias or small-study effects. The overall symmetry of the plot suggests a low likelihood of systematic bias. (B) Leave-one-out sensitivity analysis, where each individual study is sequentially removed to reassess the pooled effect estimate. Squares represent the recalculated pooled HRs after omitting each study, and horizontal lines indicate the corresponding 95% confidence intervals. Consistent pooled estimates across iterations indicate good robustness of the meta-analysis results.
