# Supplementary material for: Gender-Specific Associations Between Sex Hormones and Cardiovascular Disease: A Systematic Review and Meta-Analysis
Source: Rev Cardiovasc Med. 2026 May 13;27(5):47678. doi: 10.31083/RCM47678 (PMC13227368; doi:10.31083/RCM47678)
Supplement: Supplementary file 1 [file 2153-8174-27-5-47678-s1.zip › Supplementary Tables.docx]

Supplementary Table 1 The covariates included in multivariable-adjusted models of the eligible cohort studies

| First author/Year | Outcomes | Hormone exposure(s) | Age | Smoking | Hypertension | Diabetes | Lipids | BMI | Ethnicity | Alcohol consumption | Other covariates |
| --- | --- | --- | --- | --- | --- | --- | --- | --- | --- | --- | --- |
| Florian S. 2020 | Cardiovascular mortality | SHBG,TT,cFT,DHT,E2 | Y | Y | Y | Y | Y | Y | NR | NR | Cancer, Stroke,CRP and MI |
| Bu B. Yeap 2022 | MACE or HF | TT,SHBG,cFT | Y | Y | Y | Y | Y | Y | NR | NR | CVD and Cancer |
| R. M. Islam 2022 | MACE | DHEA, TT, | Y | Y | Y | Y | Y | Y | NR | Y | Renal dysfunction |
| D.Zhao 2018 | CVD or HF | TT, cFT,DHEA,SHBG,E2 | Y | Y | Y | Y | Y | NR | Y | NR | Study site, Hormone therapy, Waist-to-hip ratio |
| T.H. Collet 2020 | CVD | TT,E2,SHBG | Y | Y | Y | Y | Y | Y | NR | NR | Study site, Physical activity, |
| C. Meun 2018 | CVD | TT,SHBG,DHEA | Y | Y | Y | Y | Y | NR | NR | NR | Waist-hip-ratio, Hormone therapy |
| B. B. Yeap 2014 | HF | TT, cFT,DHT,E2 | Y | Y | Y | Y | Y | Y | NR | NR | Creatinine and cancer |
| D. Zhao 2020 | HF | TT, DHEA, SHBG | Y | Y | Y | Y | Y | NR | Y | NR | Waist to hip ratio, Physical activity, eGFR, |
| A. Wang 2021 | CVD or HF | TT, cFT.SHBG | Y | Y | Y | Y | Y | Y | Y | Y | Hormone therapy, Menopause, Obstructive sleep apnea. |
| Domingo A. 2009 | Cardiovascular mortality | SHBG | Y | NR | Y | NR | NR | Y | NR | NR | NR |
| Gail A. L.2010 | CAD | TT | Y | Y | NR | NR | NR | Y | NR | NR | Waist-hip ratio and Exercise |
| Y. Chen 2011 | CAD | E2, SHBG | R | Y | Y | NR | Y | Y | Y | Y | Family history of MI |
| B.B.Yeap 2021 | Cardiovascular mortality | TT, SHBG, cFT | Y | Y | Y | Y | Y | Y | NR | Y | Angina, Atrial fibrillation, COPD, Dementia, Renal impairment, Liver disease, Thyroid disease, HIV. |
| Jungeun L 2024 | HF | TT, E2 | Y | Y | YY | Y | NR | Y | NR | NR | Consumption of alcohol, Physical activity, and Education, Hormone replacement therapy, Menopausal status |
| K.Tuorila 2024 | CVD | TT, SHBG, cFT | Y | Y | Y | Y | Y | Y | NR | NR | NR |
| Katie. H 2023 | MI | TT, SHBG, cFT | Y | Y | NR | NR | NR | Y | NR | NR | NR |
| X.P. Zhan 2024 | HF | TT | Y | Y | Y | Y | Y | Y | Y | Y | Education |
| B.Hsu 2016 | Cardiovascular mortality /All-cause mortality | TT, DHT, cFT, SHBG, E2 | Y | Y | NR | NR | NR | Y | NR | NR | NR |
| Y. X. Chan 2016 | CVD | TT, cFT, DHT, E2 | Y | Y | Y | Y | Y | Y | NR | Y | Vigorous exercise, CVD or COPD history， Cancer history, Creatinine. |
| M. M. Shores2014 | CVD | TT, cFT, DHT, | Y | Y | Y | Y | Y | Y | Y | Y | Waist circumference, |
| C.Ohleeson.2011 | MACE | TT, SHBG, cFT, E2 | Y | Y | Y | Y | Y | Y | NR | NR | Physical activity |
| K.T. Khaw2007 | Cardiovascular mortality | TT | Y | Y | Y | Y | Y | Y | Y | Y | Waist-hip ratio and Education level |
| J Arnlo 2006 | CVD | TT, DHEA, E2 | Y | Y | Y | Y | Y | Y | NR | NR | NR |

Notation: Y = included in the model; NR = not reported. Abbreviations: CAD: Coronary artery disease; HF: Heart failure; CVD: Cardiovascular disease; MI: Myocardial infarction; MACE: Major adverse cardiovascular events; SHBG: Sex hormone-binding globulin; TT: Total testosterone; cFT: Calculated free testosterone; DHT: Dihydrotestosterone; E2: Estradiol; DHEA: Dehydroepiandrosterone. CRP：C-reactive protein; eGFR：estimated glomerular filtration rate; COPD：chronic obstructive pulmonary disease.

Supplementary Table 2. Circulating sex hormones, assay technologies, cardiovascular outcomes, and quality assessment of the included cohort studies.

| First author/Year | Hormones assessed | Assay Technologies | Outcomes | Quality score |
| --- | --- | --- | --- | --- |
| Florian S. 2020^[16]^ | SHBG, TT, cFT, DHT, E2 | CLIA, LC-MS/MS | Cardiovascular mortality | 8 |
| Bu B. Yeap 2022^[17]^ | TT, SHBG, cFT | LC-MS/MS | MACE/Heart failure | 8 |
| R. M. Islam 2022^[18]^ | DHEA, TT, | LC-MS/MS, CLIA | MACE | 8 |
| D.Zhao 2018^[19]^ | TT, cFT, DHEA, SHBG, E2 | RIA, CLIA | CVD/Heart failure | 8 |
| T.H. Collet 2020^[20]^ | TT, E2, SHBG | LC-MS/MS, CLIA | CVD | 8 |
| C. Meun 2018^[21]^ | TT, SHBG, DHEA | LC-MS/MS, | CVD | 8 |
| B. B. Yeap 2014^[22]^ | TT, cFT, DHT, E2 | LC-MS/MS, CLIA | Heart failure | 8 |
| D. Zhao 2020^[23]^ | TT, DHEA, SHBG | CLIA | Heart failure | 8 |
| A. Wang 2021^[24]^ | TT, cFT. SHBG | RIA, CLIA | CVD/Heart failure | 8 |
| Domingo A. 2009^[25]^ | SHBG | RIA, CLIA | Cardiovascular mortality | 8 |
| Gail A. L.2010^[26]^ | TT | RIA | CAD | 8 |
| Y. Chen 2011^[27]^ | E2, SHBG | LC-MS/MS, CLIA | CAD | 8 |
| B.B.Yeap 2021^[28]^ | TT, SHBG, cFT | LC-MS/MS, CLIA | Cardiovascular mortality | 8 |
| Jungeun L 2024^[29]^ | TT, E2 | ELISA | Heart failure | 8 |
| K.Tuorila 2024^[30]^ | TT, SHBG, cFT | LC-MS/MS, RIA | CVD | 8 |
| Katie. H 2023^[31]^ | TT, SHBG, cFT | ELISA | MI | 8 |
| X.P. Zhan 2024^[32]^ | TT | CLIA | Heart failure | 8 |
| B.Hsu 2016^[33]^ | TT, DHT, cFT, SHBG, E2 | LC-MS/MS, CLIA | Cardiovascular mortality | 8 |
| Y. X. Chan 2016[34] | TT, cFT, DHT, E2 | LC-MS/MS, CLIA | CVD | 8 |
| M. M. Shores2014^[35]^ | TT, cFT, DHT, | LC-MS/MS | CVD | 8 |
| C.Ohleeson.2011[36] | TT, SHBG, cFT, E2 | LC-MS/MS, CLIA | MACE | 8 |
| K.T. Khaw2007^[37]^ | TT | CLIA | Cardiovascular mortality | 8 |
| J Arnlo 2006^[38]^ | TT, DHEA, E2 | RIA | CVD | 8 |

Footnote: TT, total testosterone; SHBG, sex hormone-binding globulin; cFT: calculated free testosterone; E2, estradiol; DHEA, dehydroepiandrosterone; DHT, dihydrotestosterone; cFT was calculated by mass action equation. LC–MS/MS: liquid chromatography–tandem mass spectrometry; RIA: radioimmunoassay; CLIA: chemiluminescent immunoassay; ELISA: enzyme-linked immunosorbent assay. Quality was assessed using the NEWCASTLE-OTTAWA. The cohort study adopted NEWCASTLE-OTTAWA quality assessment scale The scale included three factors: patient selection, comparability of the study group, and outcome/exposure. The score of each cohort study was 0-9 (assigned by star). Getting 3 or 4 stars in the selection field, 1 or 2 stars in the comparability field, and 2 or 3 stars in the result/exposure field is defined as high quality. Select domain 2-star, comparable domain 1-star or 2-star, result/exposure domain 2 or 3-star as medium mass, domain 0 or 1-star, comparable domain 0 or 1-star, result/exposure domain 0 star or 1-star as low mass.

Supplementary Table 3 Meta-regression of the association between circulating sex hormones and CVD risk with mean age as a moderator

|  | Exposure | Outcome | Population | Moderator  (per 10 yrs) | K | Beta_  logHR (β) | 95% CI  (log HR) | p_value | Residual  I² (%) | p_value |
| --- | --- | --- | --- | --- | --- | --- | --- | --- | --- | --- |
| 1 | SHBG | CVD | Male | Mean age | 11 | -0.0004 | -0.0086-0.0078 | 0.9225 | 72.7 | <0.0002 |
| 2 | TT | CVD | Male | Mean age | 15 | 0.0015 | -0.0060-0.0090 | 0.6921 | 76.4 | <0.0001 |
| 3 | cFT | CVD | Male | Mean age | 8 | -0.0016 | -0.0087-0.0056 | 0.6716 | 53.3 | 0.0304 |
| 4 | E2 | CVD | Male | Mean age | 8 | 0.0060 | -0.0078-0.0199 | 0.3948 | 86.44 | 0.0018 |
| 5 | SHBG | CVD | Female | Mean age | 10 | 0.0116 | -0.0007-0.0339 | 0.0642 | 43.0 | 0.0177 |
| 6 | TT | CVD | Female | Mean age | 10 | -0.0105 | -0.0210-0.0001 | 0.0516 | 25.9 | 0.2073 |

β-denotes the regression coefficient for log hazard ratio (log HR) per 1-year increase in mean age at baseline in random-effects meta-regression models. Residual I² and its p value represent the proportion of between-study heterogeneity and the test for residual heterogeneity after accounting for mean age as a moderator. SHBG, sex hormone-binding globulin; TT, total testosterone; cFT, calculated free testosterone; DHT, dihydrotestosterone; E2, estradiol; DHEA, dehydroepiandrosterone; CVD, cardiovascular disease; K, number of cohorts included in each meta-regression model.

1. In a random-effects meta-regression including mean age as a continuous moderator (k = 11), age was not associated with the log hazard ratio for SHBG (β per 1-year increase = -0.0004, 95% CI -0.0086 to 0.0078; p = 0.92). Residual heterogeneity remained substantial (I² = 72.7%; QE p = 0.0002), and the proportion of between-study variance explained by age was essentially zero (R² = 0%). These findings suggest that differences in mean cohort age do not account for the observed heterogeneity in the SHBG–cardiovascular outcome association.

2. In a random-effects meta-regression using mean cohort age as a continuous moderator, the regression coefficient for age was 0.0015 (per 1-year increase; p = 0.69), with no heterogeneity explained (R² = 0%) and substantial residual heterogeneity remaining (I²=76%). These findings indicate that, within the range of mean ages across studies, there was no clear linear trend in the TT–cardiovascular outcome association with age, and mean cohort age contributed little to explaining the between-study differences in effect sizes.

3. In a random-effects meta-regression including eight male cohorts and treating mean cohort age as a continuous moderator, the regression coefficient for age was −0.0016 per 1-year increase (p = 0.67), with no between-study variance explained (R² = 0%) and residual heterogeneity remaining moderate (I² ≈ 53%). These findings indicate that, based on the currently available evidence, mean age does not materially modify the association between cFT and cardiovascular outcomes, and the cFT–CVD relationship overall appears neutral.

4. In a random-effects meta-regression using mean cohort age as a continuous moderator, the regression coefficient for age was 0.0060 per 1-year increase (p = 0.39), with no between-study variance explained (R² = 0%) and substantial residual heterogeneity remaining (I² ≈ 86%). These findings indicate that, based on the currently limited pooled data, mean age does not meaningfully modify the association between E2 and cardiovascular outcomes, and the overall E2–CVD relationship appears neutral.

5. In the random-effects meta-regression model with mean study age as a continuous moderator, the regression coefficient for age was 0.0116 (p = 0.064), explaining 3.3% of the between-study heterogeneity (R² = 3.32%), while residual heterogeneity remained significant (residual I² ≈ 43%). There was a tendency toward effect modification by age, suggesting that the positive association between SHBG and cardiovascular risk may be more pronounced at older ages; however, this evidence is borderline and exploratory, and thus insufficient to support definitive conclusions.

6. In the random-effects meta-regression model with mean study age as a continuous moderator, age showed a negative trend in its association with log(HR) (β per 1-year increase = -0.0105, p = 0.052), explaining approximately 42.1% of the between-study heterogeneity (R² = 42.1%), and the residual heterogeneity was no longer statistically significant (residual I² =25.9%, QE p = 0.21). This pattern suggests that the positive association between higher TT levels and cardiovascular risk may be more pronounced in younger women and attenuate with increasing age.

10. In a random-effects meta-regression including three female cohorts, mean age was not associated with the DHEA–cardiovascular outcome relationship (β per 1-year increase = −0.0031, p = 0.91; R² = 0%). Residual heterogeneity remained substantial (I² =89%), indicating that differences in mean cohort age did not explain the between-study variability in effect estimates and that no clear age-dependent pattern of the DHEA–CVD association could be identified.

Supplementary Table 4 Meta-regression estimates for the association between SHBG and CVD risk in men by assay technology.

| Exposure | Outcome | Population | Category | HR | 95%CI (HR) | p_value | k | β (log HR) | 95% CI (log HR) |
| --- | --- | --- | --- | --- | --- | --- | --- | --- | --- |
| SHBG | CVD | Male | CLIA | 0.74 | 0.97-1.10 | 0.8251 | 7 | -0.0211 | −0.1386-0.0964 |
| SHBG | CVD | Male | ELISA | 0.94 | 0.89-0.99 | 0.9893 | 1 | −0.0408 | −0.3126-0.2310 |
| SHBG | CVD | Male | RIA | 0.99 | 0.87-1.10 | 0.2877 | 3 | -0.0092 | −0.2308-0.2125 |

The test for subgroup differences and meta-regression analyses with assay technology as a moderator were not statistically significant, indicating that assay technology did not materially modify the association between SHBG and cardiovascular risk nor explain the observed heterogeneity. Abbreviations: SHBG, sex hormone–binding globulin; CVD, cardiovascular disease; CLIA, chemiluminescent immunoassay; ELISA, enzyme-linked immunosorbent assay; RIA, radioimmunoassay; β (log HR) and its 95% CI are regression coefficients from a mixed-effects meta-regression model with assay technology as a categorical moderator and CLIA as the reference category; positive β values indicate higher CVD risk with increasing SHBG relative to CLIA.

Supplementary Table 5 Meta-regression estimates for the association between TT and CVD risk in men by assay technology.

| Exposure | Outcome | Population | Category | HR | 95%CI (HR) | p_value | k | β (log HR) | 95% CI (log HR) |
| --- | --- | --- | --- | --- | --- | --- | --- | --- | --- |
| TT | CVD | Male | CLIA | 0.92 | 0.69-1.22 | 0.1547 | 8 | -0.0845 | -0.2062-0.0372 |
| TT | CVD | Male | ELISA | 0.82 | 0.46-1.47 | 0.0878 | 2 | -0.11287 | -0.2798-0.0225 |
| TT | CVD | Male | RIA | 0.81 | 0.26-2.46 | 0.3147 | 2 | -0.1332 | -0.4114-0.1450 |
| TT | CVD | Male | LC-MS/MS | 0.99 | 0.94-1.05 | 0.2955 | 3 | 0.0697 | -0.0700-0.2095 |

The test of meta-regression with assay technology as a categorical moderator showed significant effect modification (F(3,11) = 5.40, p = 0.016), accounting for ~94% of the between-study heterogeneity (R² = 93.8%; residual I² = 11.0%). Immunoassay-based studies (CLIA, ELISA, RIA) tended to report stronger inverse associations than LC-MS/MS-based studies, although most pairwise contrasts did not reach statistical significance because of wide confidence intervals. Abbreviations: SHBG, sex hormone–binding globulin; CVD, cardiovascular disease; CLIA, chemiluminescent immunoassay; ELISA, enzyme-linked immunosorbent assay; LC–MS/MS, liquid chromatography–tandem mass spectrometry; RIA, radioimmunoassay; β (log HR) and its 95% CI are regression coefficients from a mixed-effects meta-regression model with assay technology as a categorical moderator and CLIA as the reference category; positive β values indicate higher CVD risk with increasing SHBG relative to CLIA.

Supplementary Table 6 Meta-regression estimates for the association between E2 and CVD risk in men by assay technology.

| Exposure | Outcome | Population | Category | HR | 95%CI (HR) | p_value | k | β (log HR) | 95% CI (log HR) |
| --- | --- | --- | --- | --- | --- | --- | --- | --- | --- |
| E2 | CVD | Male | ELISA | 1.08 | 0.79-1.48 | 0.5489 | 5 | 0.0787 | -0.2363-0.3937 |
| E2 | CVD | Male | RIA | 0.62 | 0.33-1.17 | 0.1099 | 1 | -0.4792 | -1.1138-0.1553 |
| E2 | CVD | Male | LC-MS/MS | 0.99 | 0.68-1.45 | 0.9593 | 2 | -0.0079 | -0.3871-0.3713 |

However, in meta-regression with assay technology as a categorical moderator, the overall test of moderators did not reach statistical significance (F(2,5) = 2.22, p = 0.20), and assay type explained approximately 22% of the between-study heterogeneity (R² = 21.8%). These findings indicate that the association between estradiol and cardiovascular risk is overall null, with possible discrepancies between LC-MS/MS and RIA-based measurements that require confirmation in additional studies. Abbreviations: SHBG, sex hormone–binding globulin; CVD, cardiovascular disease; ELISA, enzyme-linked immunosorbent assay; LC–MS/MS, liquid chromatography–tandem mass spectrometry; RIA, radioimmunoassay; β (log HR) and its 95% CI are regression coefficients from a mixed-effects meta-regression model with assay technology as a categorical moderator and CLIA as the reference category; positive β values indicate higher CVD risk with increasing SHBG relative to CLIA.

Supplementary Table 7. Meta-regression estimates for the association between SHBG and CVD risk in women by assay technology.

| Exposure | Outcome | Population | Category | HR | 95%CI (HR) | p_value | k | β (log HR) | 95% CI (log HR) |
| --- | --- | --- | --- | --- | --- | --- | --- | --- | --- |
| SHBG | CVD | Female | CLIA | 1.02 | 0.81-1.28 | 0.8251 | 6 | 0.0225 | −0.2164- 0.2615 |
| SHBG | CVD | Female | ELISA | 1.07 | 0.98-1.17 | 0.9893 | 1 | −0.0027 | −0.4826- 0.4771 |
| SHBG | CVD | Female | LC-MS/MS | 1.33 | 0.03-55.90 | 0.8284 | 2 | 0.0451 | −0.4425-0.5327 |
| SHBG | CVD | Female | RIA | 1.02 | 0.95-1.09 | 0.2877 | 1 | 0.3050 | −0.3348-0.9447 |

The result of meta-regression with assay technology as a categorical moderator showed no evidence of effect modification (F(3,6) = 0.47, p = 0.71; R² = 0%), indicating that assay method did not materially influence the association between SHBG and cardiovascular risk nor explain the observed heterogeneity. Abbreviations: SHBG, sex hormone–binding globulin; CVD, cardiovascular disease; CLIA, chemiluminescent immunoassay; ELISA, enzyme-linked immunosorbent assay; LC–MS/MS, liquid chromatography–tandem mass spectrometry; RIA, radioimmunoassay; β (log HR) and its 95% CI are regression coefficients from a mixed-effects meta-regression model with assay technology as a categorical moderator and CLIA as the reference category; positive β values indicate higher CVD risk with increasing SHBG relative to CLIA.

Supplementary Table 8. Meta-regression estimates for the association between TT and CVD risk in women by assay technology.

| Exposure | Outcome | Population | Category | HR | 95%CI (HR) | p_value | k | β (log HR) | 95% CI (log HR) |
| --- | --- | --- | --- | --- | --- | --- | --- | --- | --- |
| TT | CVD | Female | CLIA | 1.05 | 0.98-1.12 | 0.7516 | 5 | 0.0488 | −0.3114-0.4090 |
| TT | CVD | Female | ELISA | 1.13 | 0.97-1.32 | 0.7584 | 2 | 0.0723 | −0.4770-0.6216 |
| TT | CVD | Female | LC-MS/MS | 1.23 | 0.91-1.67 | 0.4101 | 1 | 0.1601 | −0.2824-0.6026 |
| TT | CVD | Female | RIA | 1.34 | 0.08-23.7 | 0.4301 | 2 | 0.1726 | −0.3268-0.6721 |

The result of meta-regression with assay technology as a categorical moderator did not indicate significant effect modification (F(3,6) = 0.34, p = 0.80; R² = 0%), indicating that the observed association between TT and cardiovascular risk was broadly similar across different assay methods. Abbreviations: SHBG, sex hormone–binding globulin; CVD, cardiovascular disease; CLIA, chemiluminescent immunoassay; ELISA, enzyme-linked immunosorbent assay; LC–MS/MS, liquid chromatography–tandem mass spectrometry; RIA, radioimmunoassay; β (log HR) and its 95% CI are regression coefficients from a mixed-effects meta-regression model with assay technology as a categorical moderator and CLIA as the reference category; positive β values indicate higher CVD risk with increasing SHBG relative to CLIA.
